# Detection of TR34/L98H pan-azole-resistant *Aspergillus fumigatus* in poultry farm environments

**DOI:** 10.3389/ffunb.2026.1858050

**Published:** 2026-08-19

**Authors:** Bianca Gomes, Marta Dias, Renata Cervantes, Lídia Gonçalves, Elisabete Carolino, Miguel J. N. Ramos, Mónica Nunes, Ricardo Dias, Carla Viegas

**Affiliations:** 1 CE3C—Center for Ecology, Evolution and Environmental Change, Faculdade de Ciências, Universidade de Lisboa, Lisbon, Portugal; 2 H&TRC – Health & Technology Research Center, ESSL – Escola Superior de Saúde de Lisboa, Instituto Politécnico de Lisboa, Lisbon, Portugal; 3 NOVA National School of Public Health, Public Health Research Centre, Comprehensive Health Research Center, CHRC, REAL, CCAL, NOVA University Lisbon, Lisbon, Portugal; 4 Research Institute for Medicines (iMed.ULisboa), Faculty of Pharmacy, Universidade de Lisboa, Lisbon, Portugal; 5 XLab - Innovation for National Biological Resilience, Faculdade de Ciências, Universidade de Lisboa, Lisboa, Portugal

**Keywords:** *Aspergillus fumigatus*, azole-resistance profile, bedding material, *cyp51A* gene mutations, cytotoxic potential, One Health

## Abstract

**Introduction:**

Poultry farms have been recognized as environments prone to fungal contamination. However, the occurrence of azole-resistant Aspergillus fumigatus and its cytotoxic potential remain insufficiently characterized. This study aimed to characterize the occurrence, cytotoxic potential, and azole-resistance profile ofAspergillus section Fumigati in poultry farms.

**Methods:**

A total of 420 samples, including air (n = 47), electrostatic dust cloths (n = 87), bedding (n = 87), feed (n = 95), swabs (n = 87), workers’ masks (n = 2), and broiler breast (n = 15), were obtained. Fungal characterization was performed through culture-based methods (27 °C and 37 °C), followed by azole resistance screening according to EUCAST guidelines. Resistant isolates were subjected to whole-genome sequencing and a targeted analysis of cyp51A mutations. The cytotoxicity potential of fungal isolates and environmental samples was evaluated using selected cell lines representing respiratory organs (human alveolar [A549]) and detoxification organs (swinekidney [SK]/hepatocellular carcinoma [HepG2]).

**Results:**

Seven putative Aspergillus fumigatus isolates exhibited pan-azole resistance (MICs: ≥ 2 mg/L ITR/VOR; ≥ 1mg/L for POS). Four isolates carried the TR34/L98H mutation and were recovered from bedding (n = 3) and air (n = 1), suggesting the presence of resistant A. fumigatus in poultry farm matrices. Although 12% of isolates induced toxicity on both cell lines (17/145), no significant association was observed between isolate cytotoxicity and environmental sample toxicity, suggesting that additional biological and chemical components may contribute to the overall toxicological profile of farm environments.

**Discusion:**

The study highlights the occurrence of azole-resistant A. fumigatus in poultry farms and support integrated surveillance approaches addressing antifungal resistance and environmental exposure risks in poultry production.

## 1 Introduction

The global expansion of the poultry industry has significantly increased the production of poultry waste, primarily composed of animal bedding material and farm residues (e.g., feathers, excrement, and feed residues) (Gomes et al., 2022). Due to its high nutrient content, this waste is commonly used as an organic fertilizer. However, the bedding material itself can harbor a variety of pathogens, acting as a vehicle for their dissemination within poultry facilities and their surrounding environment. This dynamic underscores the critical need for a One Health approach to manage the interconnected risks to animal, human, and environmental health (Gomes et al., 2023, 2022).

The growing concern regarding antimicrobial resistance has further reinforced the importance of adopting a One Health approach when assessing microbial hazards in animal production. This approach recognizes the interconnectedness of human, animal, and environmental health, emphasizing that the emergence and dissemination of resistant microorganisms are driven by the interactions across these three domains (EFSA, 2025; Gomes et al., 2022; Verweij et al., 2020).

Among pathogens, species belonging to *Aspergillus* section *Fumigati* are characterized by their high toxicity (Viegas et al., 2021a, 2021b). The primary pathogen within section *Fumigati* is *Aspergillus fumigatus* (*sensu stricto*), the main causative agent of aspergillosis (Gniadek et al., 2017; Lamoth, 2016; Viegas et al., 2021b). Its pathogenicity is driven by several features, including the production of extremely small conidia that are easily inhaled and capable of reaching and colonizing the respiratory tract, its capacity to survive and grow in a wider range of environmental conditions, its ability to produce toxic metabolites (mycotoxins), along with other virulence factors (Gniadek et al., 2017; Hui et al., 2024; Viegas et al., 2021a).

More recently, *A. fumigatus* infections have become more difficult to cure, challenged by the rising incidence of resistance to azoles, the first-line class of antifungal agents. The most reported resistance mechanisms are typically associated with changes in the *cyp51A* gene and its promoter (EFSA, 2025; Gonçalves et al., 2020; Hsu et al., 2022). Resistant patterns are frequently composed of tandem repeat (TR) mutations in the promoter region in combination with single- or multiple-point mutations (e.g., TR_34_/L98H, TR_53_, TR_46_/Y121F/T289A) (Dladla et al., 2024; EFSA, 2025; Hsu et al., 2022). These genetic modifications compromise azole binding, reducing treatment efficacy and contributing to prolonged sickness and elevated mortality rates (Bottery et al., 2024; Dladla et al., 2024; Hui et al., 2024). This growing threat has been acknowledged by WHO (WHO, 2022), which designated *A.fumigatus* as a priority fungal pathogen, underscoring the urgent need to strengthen global surveillance and research on this topic (Hui et al., 2024; WHO, 2022).

Multiple environmental sites have been identified as hotspots for the development of azole-resistant *A. fumigatus* (ARAf). Recently, poultry farms have been recognized as potential hotspots (Gomes et al., 2022). This concern is supported by recent findings regarding potential ARAf in these settings (Gomes et al., 2025a). The common use of wood shavings, possibly treated with azoles, as animal bedding material, may promote the emergence of resistant mycobiota within poultry farms (Gomes et al., 2022). Once colonized, bedding material may represent a potential source of *A. fumigatus* conidia, which can become airborne during routine farming activities or bird movement, posing potential exposure risks for both humans and animals (Gomes et al., 2025a, 2022). These risks may also extend beyond the farm environment through the management of poultry waste and its recurrent application to agricultural fields, where fungi and other contaminants may be introduced into the environment (Gomes et al., 2025a, 2022). Given the emergence of azole resistance, further studies are required to characterize potential environmental hotspots sustaining resistant populations and elucidate their public health implications (Dladla et al., 2024; Gonçalves et al., 2020).

To characterize the biological effects associated with environmental exposure, *in vitro* evaluation through cytotoxicity analysis is widely used as a first-line screening (Dias et al., 2024; Viegas et al., 2023, 2022a). *In vitro* toxicology estimates potential health effects by assessing biological responses, such as cellular cytotoxicity or DNA damage in relevant cell models, exposed to complex mixtures of pollutants and/or contaminants. A major advantage of this approach is the ability to evaluate toxicity even when the full chemical or biological composition of the environmental sample is not fully characterized (Gomes et al., 2025b; Viegas et al., 2021b).

Recent exposure assessment studies conducted by our group reported occupational exposure to potentially azole-resistant *Aspergillus* species in environmental samples from poultry farms (Gomes et al., 2025a) and the cytotoxicity of these complex matrices (Gomes et al., 2025b). However, these studies did not evaluate the cytotoxicity potential of recovered *Aspergillus* isolates, characterize antifungal resistance mechanisms, or determine the contribution of individual isolates to the overall cytotoxicity observed in the corresponding environmental samples. Therefore, building upon the previous findings, this study aimed to perform a targeted assessment of *Aspergillus* isolates recovered from poultry farms. Specifically, it aimed to characterize *Aspergillus* section *Fumigati* prevalence, cytotoxic potential, and azole resistance profile. By screening *Aspergillus fumigatus* isolates for azole-resistance and evaluating their cytotoxic effects, this work seeks to determine the contribution of individual isolates to the overall cytotoxicity observed in the corresponding environmental samples, while providing further insight into the occurrence of azole-resistant *A. fumigatus* and its associated resistance mechanisms in poultry farms within a One Health framework.

## 2 Materials and methods

### 2.1 Research context

This study is part of a broader exploratory project assessing microbial contamination and exposure risks in poultry farms through a One Health approach (Gomes et al., 2025a, 2025b). Previous work characterized fungal contamination, mycotoxin occurrence, and the cytotoxicity of environmental samples. Building upon these findings, the present work focused on a targeted characterization of *Aspergillus* section *Fumigati* isolates, including screening for azole resistance, molecular characterization of resistance mechanisms, and assessment of cytotoxic potential.

*Aspergillus* section *Fumigati* was characterized using culture-based methods through incubation at two different temperatures (27 °C and 37 °C) to identify potential pathogenic species and was complemented with molecular detection (qPCR). Isolates that thrived at 37 °C were further evaluated for azole susceptibility, and those exhibiting phenotypic azole resistance were subsequently analyzed for resistance mechanisms in the *cyp51A* gene. Cytotoxicity assays were also performed on fungal isolates to assess their intrinsic cytotoxic potential. The cytotoxicity of these isolates was then compared with previously reported cytotoxicity results of their corresponding environmental samples (EDC, feed, and bed) (Gomes et al., 2025b), to assess whether individual isolates contributed to the overall cytotoxicity of each environmental sample.

Since the corresponding environmental samples had also been previously screened for mycotoxins, the present study further investigated the association between mycotoxin occurrence and cytotoxicity in environmental samples.

Environmental samples were screened for 38 mycotoxins, including aflatoxins (AFs), fumonisins (FBs), trichothecenes (TRCs), ochratoxins (OTs), zearalenone (ZEN), and gliotoxin. The complete list of targeted mycotoxins is provided in **Supplementary Table 1**. Samples (EDC, feed, and bed) were extracted using an acetonitrile/water/acetic acid solution (79:20:1, v/v/v) and centrifuged. Mycotoxin analysis was performed using a Nexera high-performance liquid chromatograph (Shimadzu, Tokyo, Japan) coupled to a QTRAP 5500 mass spectrometer (Sciex, Foster City, CA, USA), following a preestablished LC-MS/MS method (Viegas et al., 2020a). The complete analytical protocol, including sample preparation, chromatographic conditions, quality control procedures, and limits of detection and quantification for each analyte, has been previously described (Gomes et al., 2025b). In this study, mycotoxin occurrence was treated as a binary variable (0 = not detected; 1 = detection of at least one mycotoxin) to investigate its association with cytotoxicity in environmental samples.

### 2.2 Sampling approach and material collection

This study was conducted in five poultry farms, located on Madeira Island, Portugal. Each farm comprised between two and four poultry pavilions (PP), resulting in a total of 14 PP. Briefly, all farms were characterized by small-scale broiler production with up to three workers per farm. Each worker was responsible for daily monitoring of bird health and farm conditions. Poultry pavilions accommodated approximately 6,200 to 9,180 broilers, with areas ranging from 410 to 512 m^2^. Broilers were reared for approximately 35 days on wood shavings bedding, which was introduced at the beginning of the production cycle and maintained throughout the entire rearing period. Natural ventilation was the primary ventilation system used. At the end of each production cycle (approximately 35 days), broilers were transported to the slaughterhouse for processing. The bedding material (poultry waste) was subsequently removed from the pavilions, and the empty facilities were cleaned and disinfected using a bactericidal, fungicidal, virucidal, and sporicidal solution. Poultry waste was mainly reused as an organic fertilizer in agriculture.

Samples were collected from 14 PPs throughout four stages of the production cycle as follows: stage 0 related to cleaned and disinfected empty pavilions, and stages 1–3, corresponding to the first, second, and third weeks of the poultry growth cycle, respectively. The sampling frequency was performed weekly, with one sample of each method collected per PP. The sampling campaign covered two consecutive production cycles per PP during summer and winter, with mean indoor temperatures of 26.46 °C ± 3.79 °C and 22.10 °C ± 4.14 °C, respectively.

When considering each pavilion (*n* = 14), one indoor air sample was collected through a MAS-100 air sampler (Millipore, Billerica, MA, USA) mounted with polycarbonate filters (0.2 μm pore; 47 mm; Merck Millipore Ltd., Cork, Ireland) (Lanier et al., 2010). Air sampling was set at 100 L/min for 5 minutes at the center of each PP. At the same location, an electrostatic dust cloth (EDC, Swiffer, Portugal) was positioned horizontally at a height of 1.5 m above the floor, and it was replaced weekly. A surface swab was performed on the pavilion’s walls using a square stencil of 10 cm × 10 cm area, previously disinfected with a 70% alcohol solution. Furthermore, a composite sample (10 g) of animal bedding material (wood shavings) and feed (from the feeders) was obtained. Additionally, sealed feed samples (10 g) of all feed types (*n* = 3) used during the production cycle were provided (Gomes et al., 2025a).

Additional matrices were recovered to expand the scope of *A. fumigatus* assessment in poultry farms. Workers’ filtering respiratory protection devices (FRPD) (*n* = 2) were included, reflecting the availability of workers who wore respiratory protection, mainly during disinfection procedures. Regarding biological samples, the sampling design was adapted from Commission Regulation (EC) No. 2073/2005 on microbiological criteria for foodstuffs (European Commission, 2005), which establishes a standardized sampling scheme for poultry carcasses. Accordingly, 15 broiler carcasses were collected per poultry farm at the slaughterhouse. Breast tissues were pooled from three carcasses to obtain five composite samples (10 g each) of broiler breast per farm.

All samples were placed in sterilized bags and transported under refrigerated conditions (0 °C–4 °C) to the laboratory for further analyses (Viegas et al., 2022a). Overall, 420 samples were collected, comprising indoor air samples (*n* = 47), EDCs (*n* = 87), feed (*n* = 95), bedding material (*n* = 87), surface swabs (*n* = 87), workers’ FRPD (*n* = 2), and broiler breast (*n* = 15). The distribution of samples across the five poultry farms (A–E), comprising a total of 14 PP, is summarized in **Table 1**.

**Table 1:** Distribution of samples according to matrix and the number (*N*) of pavilions comprising the five poultry farms.

Parameter/sample	Farm A	Farm B	Farm C	Farm D	Farm E	Total
Pavilions (*N*)	4	4	2	2	2	14
Air	13	13	7	7	7	47
EDC	26	26	7	14	14	87
Feed	28	26	11	16	14	95
Bed	26	26	7	14	14	87
Swabs	26	26	7	14	14	87
FRPD	Na	Na	Na	Na	2	2
Broilers breast	Na	5	Na	5	5	15
Total	119	122	39	70	70	420

FRPD, filtering respiratory protective devices; Na, not evaluated.

### 2.3 Samples extraction

Sample extraction and mycobiota characterization were described in detail in previous research (Gomes et al., 2025a, 2025b). Briefly, samples were processed through elution in 0.1% Tween 80 saline (0.9% NaCl) under orbital shaking. Bed, feed, EDC, polycarbonate filters (PC), and swabs were washed in the respective volumes: 90, 90, 20, 5, and 1 mL of elution solution. Following orbital shaking at the respective conditions: 100 rpm, 30 min (bed, feed); 250 rpm, 30 min (EDC, swabs); and 500 rpm, 15 min (PC). FRPD were eluted in 10 mL of 0.1% Tween 80 saline (0.9% NaCl) by orbital shaking at 250 rpm, 30 min following the procedure applied by Dias et al. (2024). Breast samples were homogenized in 90 mL of 0.1% Tween 80 saline (0.9% NaCl) using a laboratory homogenizer (Smasher) for 2 min. The procedure was adapted from previously described methodologies for microbiological analysis of poultry samples (Kim and Yim, 2016; Morshdy et al., 2021).

### 2.4 *Aspergillus* section *Fumigati* characterization

Sample aliquots (150 µL) were inoculated onto Dichloran-glycerol agar (DG18). This medium was chosen to improve fungal isolation, preventing the spread of species with rapid growth rates (Gomes et al., 2025b; Viegas et al., 2021a; S.Viegas et al., 2020). All plates were incubated at 27 °C and subsequently at 37 °C to identify thermotolerant species capable of growing at human body temperature, a critical indicator for pathogenicity (Hernández-Benítez et al., 2025; Sueiro-Olivares et al., 2015). After 5–7 days, fungal colonies were quantified as colony-forming units (CFU), and concentrations were obtained as CFU/m^3^ (air), CFU/m^2^ (swabs, FRPD), CFU/m^2^/day (EDC), and CFU/g (bed, feed, and broiler breast). *Aspergillus* section *Fumigati* species were characterized based on standard phenotypic characteristics (Hoog et al., 2016).

From all isolates recovered (*n* = 253), isolates that grew at 37 °C (*n* = 173) were selected for further analysis.

### 2.5 Assessment of antifungal resistance

#### 2.5.1 Isolates selection

*Aspergillus* section *Fumigati* isolates (*n* = 173), previously cultured at 37 °C, were sub-cultured on Potato Dextrose Agar (PDA) at 50 °C to select putative *Aspergillus fumigatus* isolates (*n* = 164), as thermotolerance is an essential characteristic of this species to differentiate it from morphologically similar species (cryptic species) within the section *Fumigati* (Frías-De-León et al., 2023; Lamoth, 2016; Sugui et al., 2015). Putative *A. fumigatus* isolates were further analyzed for azole resistance. In parallel, *Aspergillus* section *Fumigati* isolates (*n* = 173) were evaluated for their cytotoxic potential.

#### 2.5.2 Azole-resistance screening

Putative *Aspergillus fumigatus* isolates (*n* = 164) were screened for azole resistance through the azole-containing agar plate method (European Committee on Antimicrobial Susceptibility Testing (EUCAST) E.Def 10.1), as previously described by Guinea et al. (2019). Briefly, inoculum suspensions were prepared from pure colonies according to the adaptation proposed by Serrano-Lobo et al. (2024), in which suspensions are neither filtered nor adjusted. Conidial suspensions (25 µL) were inoculated into four-well RPMI-1640 agar plates (Sigma-Aldrich, Spain), with the following concentrations: 4 mg/L itraconazole (ITR), 2 mg/L voriconazole (VOR), and 0.5 mg/L posaconazole (POS), respectively. Plates were incubated at 37 °C for 48 h. *Aspergillus fumigatus* isolates exhibiting visual growth on any of the azole-supplemented wells were considered potentially resistant and were further selected for microdilution susceptibility testing.

#### 2.5.3 Broth microdilutions susceptibility testing

Putative azole-resistant isolates identified by the azole-containing agar plate method (EUCAST E.Def 10.1) were subsequently confirmed using the EUCAST broth microdilution reference method (EUCAST E.Def 9.4), following the pre-established protocol from Guinea et al. (2022). Antifungals (ITR, VOR, POS) (Sigma-Aldrich, Spain) were tested at concentrations ranging from 8 to 0.016 mg/L. Conidial suspensions were prepared from fresh mature colonies (3–5-day-old) and adjusted to the recommended inoculum concentration (1–2.5 × 10^5^ CFU/mL) before inoculation onto microdilution plates. Each isolate was tested in duplicate wells, and plates were incubated at 37 °C for 48 h. The minimum inhibitory concentrations (MICs) were defined as the lowest concentration of the antifungal agent (mg/L) that inhibits visible fungal growth. *Aspergillus flavus* ATCC 2004304 and *Aspergillus fumigatus* ATCC 2004305 were included as quality control strains to validate the MICs. MIC values were interpreted according to EUCAST clinical breakpoints (EUCAST clinical breakpoints (version 12.0 , EUCAST, 2025)). Isolates were considered resistant when MICs exceeded 1 mg/L for ITR and VOR and 0.125 mg/L for POS. Pan-azole-resistant isolates were defined as those showing resistance to all three azoles according to these breakpoints. Additional methodological details are presented in **Supplementary Table 2**.

#### 2.5.4 DNA extraction and whole-genome sequencing

Azole-resistant isolates were recovered from pure colonies, and DNA was extracted following the GES protocol (guanidinium thiocyanate-based), with minor modifications (Pitcher et al., 1989). After extraction, DNA was quantified using a Qubit Fluorometer (v1.01) with the Qubit dsDNA High Sensitivity Assay Kit, and purity was assessed using a NanoDrop ND-1000 spectrophotometer.

#### 2.5.5 Bioinformatic analysis and detection of *cyp51A* mutations

Whole Genome Sequencing was performed using Oxford Nanopore technology. Nanopore reads were basecalled with Dorado v7.9.8 using the high-accuracy 400 bps model and assessed with NanoPlot v1.42.0 (Coster and Rademakers, 2023). To minimize coverage-driven bias across samples, FASTQ files were downsampled with seqtk v1.5 to match the read count of the lowest-depth sample. Normalized reads were aligned to the reference genome AF293 (GCF_000002655.1) with minimap2 v2.30 using the parameters -a -x map-ont (Li, 2018). Alignments were sorted and indexed with samtools v1.22.1 (Li et al., 2009). Variants were called with Clair3 v1.2.0 using ONT settings (--platform=“ont”) and the model r1041_e82_400bps_sup_v500 (Zheng et al., 2021), enabling all contigs (--include_all_ctgs) and applying thresholds of QUAL ≥ 20 (--qual=20) and minimum coverage ≥ 10 (--min_coverage=10). Variants were further annotated for predicted functional effects using SnpEff v5.2 (Cingolani et al., 2012), with the default parameters, including annotation of putative promoter regions up to 5,000 bp upstream of annotated genes. Variants of interest were further inspected in IGV (v2.16.2) (Robinson et al., 2011), using the reference genome and annotation, together with the read alignments for each sample. Potential contamination was assessed using Kraken2 v2.1.6 against the k2_pluspfp_20251015 database (Wood et al., 2019).

### 2.6 Cytotoxicity evaluation of isolates

Cytotoxicity of *Aspergillus* section *Fumigati* isolates was assessed in selected cell lines, representing the respiratory tract (human alveolar lung carcinoma cell line [A549], ATCC^®^ CCL-185™) and a detoxification organ (human hepatocellular carcinoma cell line [HepG2], ATCC HB-8065). The A549 cell line represents the primary site of exposure to airborne fungal spores, whereas HepG2 represents the potential systemic effects of mycotoxin exposure (Wright et al., 2001). From the 173 *Aspergillus* section *Fumigati* isolates grown at 37 °C, 145 were successfully re-isolated from pure colonies and were evaluated for their cytotoxicity. The cytotoxicity assay was performed using the 3-(4,5-dimethylthiazol-2-yl)-2,5-diphenyltetrazolium bromide (MTT) assay, following previously established protocols (Gaspar et al., 2016). Briefly, cells were seeded in 96-well plates at a density of 1 × 10^4^ cells/well using RPMI 1640 culture medium (Gibco Life Technologies, Carlsbad, CA, USA) supplemented with 10% fetal bovine serum, 100 units/mL penicillin G (sodium salt), 100 μg/mL streptomycin sulfate, and 2 mM l-glutamine, and incubated at 37 °C for 24 h. Subsequently, 10 μL of fungal suspension, prepared from pure colonies and adjusted to 0.5 McFarland standard, was added to each well containing preseeded cells. In the negative control, cells were incubated with culture medium, and in the positive control, sodium dodecyl sulfate (SDS) (0.1 mg/mL, Sigma) was added to promote cell lysis. After 24 h of exposure, the medium was replaced with fresh medium containing 0.5 mg/mL of MTT dye. Following MTT incubation, absorbance was measured at 570 nm in a microplate reader (FLUOstar Omega, BMGLabtech, Germany). Cell viability (%) was calculated by normalizing the absorbance values against the negative control. A reduction in cell viability below 70% was considered indicative of a cytotoxic effect (Gaspar et al., 2016). Detailed experimental conditions, including assay parameters and cell viability calculation (%), are provided in **Supplementary Table 3**.

### 2.7 Statistical analysis

Data analysis was performed using IBM SPSS Statistics version 30.0 for Windows. Descriptive statistics were presented as frequencies and percentages (*n*, %). Differences in fungal detection rates across stages were assessed using the Chi-square test, and Fisher–Freeman–Halton’s test was applied when more than 20% of cells had expected counts below 5 or when any expected count was less than 1. Differences in detection rates between methods were assessed using McNemar’s test. To assess whether the cytotoxic effects of the isolates were associated with the overall cytotoxicity, the results were dichotomized (0 = no cytotoxic effects; 1 = cytotoxic) and compared (environmental sample/fungal isolate) on the A549 cell line, as this was the model used to test both sample types. The strength of agreement was quantified using Cohen’s kappa coefficient (κ). Associations between cytotoxicity and mycotoxin detection (0 = not detected; 1 = detected), previously described in environmental samples (Gomes et al., 2025b), were initially examined using Chi-square tests. Effect sizes were expressed as Cramer’s *V*. Odds ratios (OR) with 95% confidence intervals (CI) were also calculated. To account for the clustered sampling design, generalized estimating equations (GEE) with a binomial distribution and a logit link function were additionally applied. Poultry farms were included as the clustering variable, while matrix type, production stage, and season were included as explanatory variables, as appropriate. OR and corresponding 95% CI were estimated. Given the limited number of poultry farms (*n* = 5), these analyses should be interpreted as exploratory. Statistical significance was set at *p* < 0.05.

**Figure 1:**
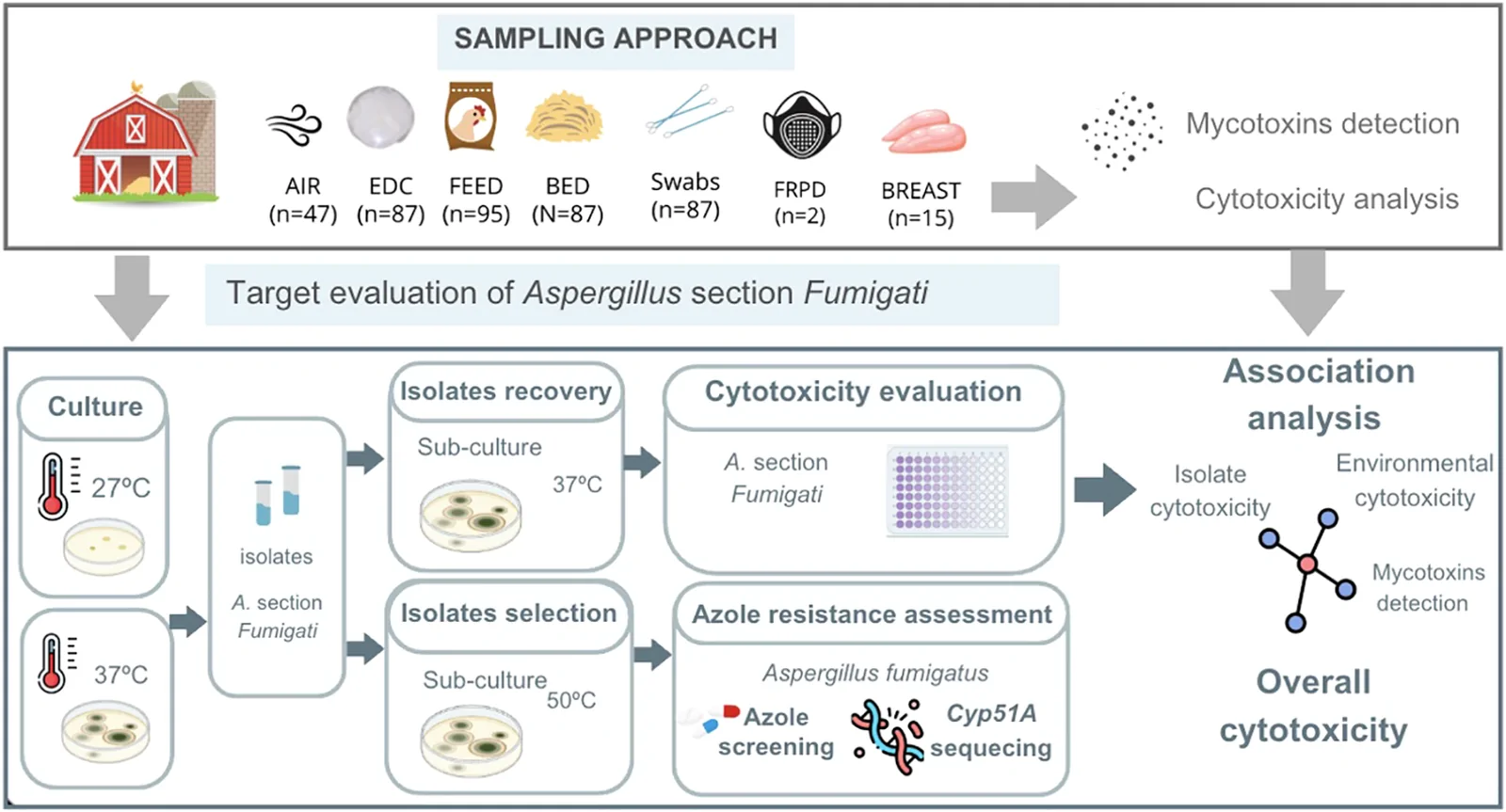
Study design and analyses performed.

A schematic representation of the study design is presented in **Figure 1**.

## 3 Results

First, we characterized the prevalence of *Aspergillus* section *Fumigati* across different matrices (air, EDC, feed, bed, swabs, FRPD, and broilers’ breasts) and production stages (0–3). Thermotolerant isolates were subsequently evaluated for azole resistance and cytotoxic potential.

### 3.1 Prevalence of *Aspergillus* section *Fumigati* across matrices

**Figure 2:**
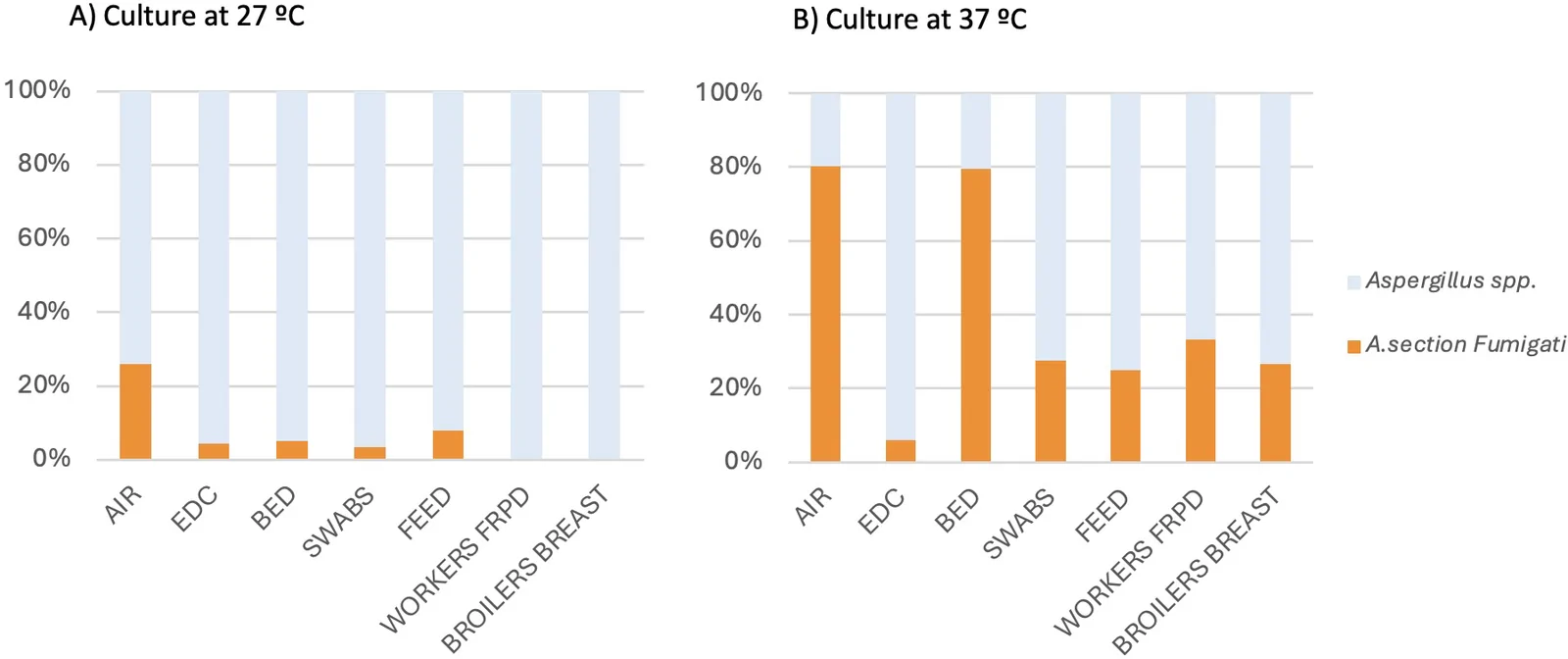
Distribution of *Aspergillus* sections in each matrix incubated at 27 °C **(A)** and 37 °C **(B)** in samples collected from poultry pavilions, workers’ filter respiratory protective devices (FRPD), and slaughterhouse (broilers’ breasts).

At 27 °C, section *Fumigati* represented 26.04% of total *Aspergillus* spp. recovered from air samples (1.50 × 10^2^ out of 5.76 × 10^2^ CFUs). Lower relative abundances were observed for the remaining matrices, as follows: feed (8.04%; 3.71 × 10^2^/4.62 × 10^3^ CFUs), bedding (5.00%; 9.40 × 10^2^/5.65 × 10^3^ CFUs), EDC (4.45%; 7.50 × 10^2^/1.69 × 10^4^ CFUs), and swabs (3.49%; 1.80 × 10^5^/5.16 × 10^6^ CFUs) (**Figure 2a**).

At 37 °C, section *Fumigati* was predominant in air (80.24%; 2.60 × 10^2^/3.24 × 10^2^ CFUs) and bedding (79.43%; 5.91 × 10^3^/7.44 × 10^3^ CFUs). This was followed by FRPD (33.33%; 5.0 × 10^1^/1.50 × 10^2^ CFUs), swabs (27.59%; 3.20 × 10^4^/1.60 × 10^5^ CFUs), feed (24.90%; 5.10 × 10^2^/2.05 × 10^3^ CFUs), broiler breast (26.67%; 2.00 × 10^2^/7.50 × 10^2^ CFUs), and EDC (5.92%; 5.30 × 10^2^/8.93 × 10^3^ CFUs) (**Figure 2b**).

### 3.2 *Aspergillus* section *Fumigati* prevalence throughout the production cycle

Section *Fumigati* was detected in all matrices (air, EDC, bed, swabs, and feed) in all production stages (0–3), at 37 °C (95.78%, 386 out of 403 samples), whereas the lowest values were obtained at 27 °C (14.64%, 59 out of 405) (**Table 2**).

**Table 2:** Detection frequency of *Aspergillus* section *Fumigati* across production stages (0, 1, 2, and 3 weeks) using culture-based methods (inoculation at 27 °C/37 °C).

Matrix	Stage	Culture detection % (n/N)	
		27 °C	37 °C
Air	0	40.00% (2/5)	80.00% (4/5)
	1	21.40% (3/14)	50.00% (7/14)
	2	35.70% (5/14)	71.40% (10/14)
	3	42.90% (6/14)	78.60% (11/14)
EDC	0	11.10% (1/9)	100% (9/9)
	1	23.10% (6/26)	100% (26/26)
	2	7.70% (2/26)	100% (26/26)
	3	23.10% (6/26)	100% (26/26)
Bed	0	11.10% (1/9)	100% (9/9)
	1	0% (0/26)	100% (26/26)
	2	0% (0/26)	100% (26/26)
	3	11.54% (3/26)	100% (26/26)
Swabs	0	22.20% (2/9)	100% (9/9)
	1	19.23% (5/26)	100% (26/26)
	2	11.55% (3/26)	100% (26/26)
	3	19.23% (5/26)	96.15% (25/26)
Feed	0	11.76% (2/17)	100% (17/17)
	1	3.85% (1/26)	100% (26/26)
	2	11.54% (3/26)	100% (26/26)
	3	11.54% (3/26)	96.15% (25/26)
Total	(0–3)	14.64% (59/403)	95.78% (386/403)

*n*, total number of positive samples; *N*, total number of samples.

Overall, *A.* section *Fumigati* did not differ significantly across production stages (culture at 27 °C: *χ*^2^(3) = 3.986, *p* = 0.263; culture at 37 °C: *χ*^2^(3) = 1.969, *p* = 0.579). In contrast, detection rates varied significantly across inoculation temperatures (McNemar = 318.40, *df* = 1, *p* < 0.001) (**Supplementary Table 4**).

#### 3.2.1 Azole-resistance screening of recovered isolates and *cyp51A* mutations

Screening based on the agar-plate method identified seven (out of 164) potentially resistant isolates, six from bed and one from air samples (**Supplementary Table 5**). These were confirmed through the broth microdilution method. The MIC values ranged from 2 to 8 mg/L on ITR, 2 to 4 mg/L on VOR, and 1 to 2 mg/L on POS (**Table 3**). All seven isolates evidenced pan-azole resistance (ITR, VOR, and POS) and were selected for whole-genome sequencing analysis.

**Table 3:** Growth of putative azole-resistant *A. fumigatus* isolates on azole screening media, minimal inhibitory concentrations (MIC) for itraconazole (ITR), voriconazole (VOR), and posaconazole (POS), and mutations identified in the *cyp51A* gene.

Isolate	Matrix	Azole screening media			MIC (mg/L)			*cyp51A* mutations	
		ITR	VOR	POS	ITR	VOR	POS	Promoter region	Coding region
160CME3	Air	−	+	+	4	4	2	**TR34**: four indels^*^	**L98H,** F46Y, M172V, N284T, D255E, E427K, C454C, L358L, G89G
157DB3	Bed	+	+	+	2	2	1	Four indels^*^	F46Y, M172V, N284T, D255E, E427K, C454C, L358L, G89G
226BB2	Bed	+	+	+	2	2	1	**TR34**: four indels^*^	**L98H**, F46Y, M172V, N284T, D255E, E427K, C454C, L358L, G89G
229AB2	Bed	+	+	+	2	2	1	**TR34**: four indels^*^	**L98H**, F46Y, M172V, N284T, D255E, E427K, C454C, L358L, G89G
245AB1	Bed	+	−	+	4	2	1	Four indels^*^	F46Y, M172V, N284T, D255E, E427K, C454C, L358L, G89G
268BB3	Bed	+	+	+	8	4	2	Four indels^*^	F46Y, M172V, N284T, D255E, E427K, C454C, L358L, G89G
269AB3	Bed	+	+	+	8	4	2	**TR34**: four indels^*^	**L98H**, F46Y, M172V, N284T, D255E, E427K, C454C, L358L, G89G

Mutations shown in bold indicate classical resistance-associated mutations described in the literature.

“−”, negative (no growth); “+”, relevant growth (growth similar to positive control).

^*^c.-1548_-1545delCTGG; c.-1625_-1624insT; c.-914_-913insT; c.-2380delT.

Whole-genome sequencing (WGS) generated high-quality datasets across the seven putative azole-resistant *A. fumigatus* isolates, producing 13.02 million reads and 48.76 Gb of sequence data. Sequencing depth ranged from 168.7 × to 313.7 ×, with 93%–95% genome fraction coverage and 96%–98% of reads successfully mapped against the *A. fumigatus* Af293 reference genome. Taxonomic classification using Kraken2 confirmed the predominance of *A. fumigatus* reads across all datasets (94.7%–96.2%), with less than 1% of reads remaining unclassified. A summary of WGS quality metrics for each isolate is provided in **Supplementary Tables 6**, **S7**.

Analysis of the *cyp51A* gene identified the TR34/L98H mutation in four isolates. The remaining three isolates lacked tandem repeats in the *cyp51A* promoter region but had the mutations: F46Y, M172V, N284T, D255E, E427K. Furthermore, four indels upstream of the promoter were identified (c.-1548_-1545delCTGG; c.-1625_-1624insT; c.-914_-913insT; c.-2380delT) (**Table 3**).

### 3.3 Isolates cytotoxicity assessment

From all the tested section *Fumigati* thermotolerant isolates (*n* = 145), 46.20% (67/145) induced toxicity on HepG2 cells, reducing cell viability to as low as 34%. Regarding A549 cells, 16.55% (24/145) of isolates reduced cell viability to as low as 57%. Of note, 12% of isolates (17/145) induced toxicity on both cell lines. When comparing matrices, isolates recovered from air samples showed the highest frequency of cytotoxicity in HepG2 cells (65.8%, 25/38), whereas cytotoxicity in A549 cells was generally less frequent across all matrices, with the highest proportion observed among isolates recovered from swabs (25.7%, nine of 35). Cytotoxicity results of each isolate are presented in **Supplementary Table 8**, and a summary of cytotoxicity outcomes according to matrix and production stage is provided in **Supplementary Table 9**.

### 3.4 Correlation and comparison analysis

#### 3.4.1 Isolates vs. environmental sample cytotoxicity

Cytotoxicity of fungal isolates was further compared with previously obtained cytotoxicity data from their corresponding environmental samples (EDC, feed, and bed) (Gomes et al., 2025b). Cytotoxicity outcomes were dichotomized (0 = no cytotoxic effects; 1 = cytotoxic) and are presented in detail in **Supplementary Table 10**. For this comparison, a total of 65 isolates obtained from EDC, feed, and bed were eligible for comparison, corresponding to samples for which cytotoxicity was assessed using the same cell line (A549).

Overall, 76.90% (50/65) of environmental samples induced cytotoxicity, whereas 10.77% (7/65) of the corresponding *Aspergillus* section *Fumigati* isolates exhibited cytotoxic effects.

No association was detected between cytotoxicity in environmental samples and that in fungal isolates (*χ*^2^ (1) = 0.342, *p* = 0.559 for A549, with a very small effect size (Phi = 0.072) and low concordance between the two types of samples (Kappa = 0.027) (**Table 4**). The pattern of discordance, dominated by cytotoxic environmental samples with noncytotoxic isolates, suggests that cultivable fungi alone are not the main contributors to the observed toxic profile in environmental samples.

**Table 4:** Association between cytotoxicity in environmental samples and fungal isolates.

Fungal isolated				Test statistic			Effect size^a^	Agreement analysis	
Environmental samples		Noncytotoxic	Cytotoxic	Chi-square		*df*	*p*-value	Cohen’s Kappa coefficient	*p*-value
Noncytotoxic	*n*	14	1		0.342	1	0.559	0.0072	0.027	0.559
	% within A549-isolated	24.10%	14.30%							
Cytotoxic	*n*	44	6							
	%within A549-isolated	75.90%	85.70%							

Results of the Chi-square test, effect size, and agreement analysis.

a Cramer’s *V*.

To account for the clustered sampling design, supplementary GEE analyses were performed using poultry farms as the clustering factor and matrix, production stage, and season as explanatory variables. For fungal isolates, no significant associations were observed between cytotoxicity and matrix (Wald *χ*^2^ = 0.306, *p* = 0.580), season (Wald *χ*^2^ = 0.058, *p* = 0.810), or production stage (Wald *χ*^2^ = 0.270, *p* = 0.874) (**Supplementary Table 11**). For environmental samples, cytotoxicity was significantly associated with matrix type (Wald *χ*^2^ = 710.534, *p* < 0.001) and production stage (Wald *χ*^2^ = 184.256, *p* < 0.001), but not with season (Wald *χ*^2^ = 0.489, *p* = 0.484) (**Supplementary Table 12**). Specifically, bed samples showed higher odds of cytotoxicity than feed samples (OR = 6.87, 95% CI: 3.00–15.74), whereas EDC samples showed lower odds (OR = 0.13, 95% CI: 0.03–0.56). Stage 2 samples also showed higher odds of cytotoxicity compared with stage 3 (OR = 3.57, 95% CI: 2.71–4.70) (**Supplementary Table 10**).

#### 3.4.2 Mycotoxin detection vs. cytotoxicity of environmental samples

A total of 38 mycotoxins were screened in environmental samples (EDC, feed, and bed). Among these, DON, FBs (FB1, FB2), and ZEA were detected in all three matrices. Other mycotoxins were detected in specific matrices, such as STER and roquefortine C (ROQ-C), which were detected in both feed and bedding material. The detected mycotoxins and concentration ranges (ng/g) are presented in **Supplementary Table 1**. Their frequencies (%) in the environmental samples have been previously reported (Gomes et al., 2025b).

Using the previously generated data, the present study investigated the association between mycotoxin occurrence and cytotoxicity in environmental samples. Mycotoxin occurrence was dichotomized (0 = not detected; 1 = detected) and compared with cytotoxicity outcomes (0 = no cytotoxic effects; 1 = cytotoxic) for the selected cell lines (SK cells; A549 cells).

A total of 250 samples were eligible for comparison. Of these, 67.60% (169/250) induced cytotoxicity in SK cells, 64.40% (161/250) induced cytotoxicity in A549 cells, and 62.89% (157/250) of samples showed detectable mycotoxins. These results are detailed in **Supplementary Tables 13**, **S14**.

The association between mycotoxin detection and cytotoxicity varied according to the cell line. For A549 cells, no significant association was observed in the bivariate analysis (*χ*^2^(1) = 1.787, *p* = 0.181; Cramer’s *V* = 0.085), although cytotoxic samples showed a slightly higher frequency of mycotoxin detection (65.8%) than non-cytotoxic samples (57.3%) (**Table 5**). Consistently, in the GEE model accounting for clustering within poultry farms and adjusting for matrix type and production stage, mycotoxin detection was not significantly associated with cytotoxicity (OR = 1.21, 95% CI: 0.68–2.15; *p* = 0.512). However, matrix type and production stage remained significant predictors of cytotoxicity, with bed samples showing higher odds of cytotoxicity than feed samples (OR = 3.04, 95% CI: 1.49–6.21; *p* = 0.002), whereas EDC samples showed lower odds (OR = 0.20, 95% CI: 0.13–0.30; *p* < 0.001) (**Table 5**).

**Table 5:** Association between cytotoxicity and mycotoxin detection in environmental samples.

Environmental samples		Mycotoxin detection - environmental samples		Test statistic			Effect size^a^	Odds Ratio	95% Confidence Interval for OR	
		Non-detected	Detected	Qui-Square	df	p			Lower bound	Upper bound
A549 Environmental samples	Non-cytotoxic	38	51	1.787	1	0.181	0.085	1.436	0.844	2.443
	Cytotoxic	55	106							
SK Environmental samples	Non-cytotoxic	40	41	7.612	1	0.006^b^	0.174	2.135	1.240	3.677
	Cytotoxic	53	116							

Results of the Chi-square test, effect size, and odds ratio.

a Cramer’s *V*.

b *p*-values < 0.05 were considered statistically significant.

For SK cells, a significant association was detected between cytotoxicity and mycotoxin detection in the bivariate analysis (*χ*^2^(1) = 7.612, *p* = 0.006; Cramer’s *V* = 0.174), indicating that cytotoxic samples were approximately twice as likely to contain detectable mycotoxins as non-cytotoxic samples (OR = 2.14, 95% CI: 1.24–3.68) (**Table 4**). This association remained significant after accounting for poultry farm clustering and adjusting for matrix type and production stage in the GEE model. Samples with detectable mycotoxins showed approximately 1.8-fold higher odds of cytotoxicity than samples without detectable mycotoxins (OR = 1.81, 95% CI: 1.37–2.39; *p* < 0.001). In the adjusted model, EDC samples also showed lower odds of cytotoxicity than feed samples (OR = 0.29, 95% CI: 0.22–0.37; *p* < 0.001), while stage 0 samples showed higher odds of cytotoxicity than stage 3 samples (OR = 3.75, 95% CI: 2.99–4.69; *p* < 0.001) (**Table 6**).

**Table 6:** Results of generalized estimating equations (GEE) with a binomial distribution and logit link function for cytotoxicity in A549 cells and SK cells with respect to mycotoxin detection.

Model	Parameter	*B*	Std. error	95% Wald confidence interval		Odds ratio	95% Wald confidence interval for odds ratio		Hypothesis test		
				Lower	Upper		Lower	Upper	Wald Chi-square	*df*	Sig.
Dependent variable: cytotoxicity in A549 cells Model: intercept matrix, stage, mycotoxin detection	(Intercept)	1.138	0.246	0.656	1.620	3.121	1.928	5.052	21.434	1	0.000
	(Matrix = bed)	1.113	0.365	0.399	1.827	3.042	1.490	6.213	9.329	1	**0.002**
	(Matrix = EDC)	− 1.620	0.206	− 2.022	− 1.218	0.198	0.132	0.296	62.335	1	**0.000**
	(Matrix = Feed)	0^a^				1					
	(Stage = 0)	− 0.707	0.131	− 0.962	− 0.452	0.493	0.382	0.636	29.560	1	**0.000**
	(Stage = 1)	− 0.457	0.263	− 0.971	0.058	0.633	0.379	1.060	3.027	1	0.082
	(Stage = 2)	− 0.233	0.059	− 0.348	− 0.117	0.793	0.706	0.889	15.584	1	**0.000**
	(Stage = 3)	0^a^				1					
	(Mycotoxin detection = 0)	0.192	0.293	− 0.382	0.766	1.212	0.683	2.150	0.431	1	0.512
	(Mycotoxin detection = 1)	0^a^				1					
Dependent variable: cytotoxicity in SK cells Model: intercept, matrix, stage, mycotoxin detection	(Intercept)	1.307	0.225	0.865	1.749	3.695	2.375	5.746	33.632	1	0.000
	(Matrix = Bed)	− 0.315	0.258	− 0.821	0.191	0.730	0.440	1.210	1.492	1	0.222
	(Matrix = EDC)	− 1.251	0.133	− 1.512	− 0.990	0.286	0.221	0.371	88.494	1	**0.000**
	(Matrix = Feed)	0^a^				1					
	(Stage = 0)	1.321	0.115	1.096	1.545	3.746	2.993	4.689	132.936	1	**0.000**
	(Stage = 1)	0.591	0.303	− 0.003	1.184	1.805	0.997	3.268	3.803	1	0.051
	(Stage = 2)	0.010	0.355	− 0.687	0.706	1.010	0.503	2.027	0.001	1	0.978
	(Stage = 3)	0^a^				1					
	(Mycotoxin detection = 0)	− 0.591	0.142	− 0.870	− 0.313	0.554	0.419	0.731	17.342	1	**0.000**
	(Mycotoxin detection = 1)	0^a^				1					

Statistically significant associations (*p* < 0.05) are indicated in bold.

a Set to zero because this parameter is redundant.

## 4 Discussion

In poultry production, *A. fumigatus* contamination has been recognized as a significant concern, with far-reaching implications for humans, animals, and environmental health (Gomes et al., 2023; Melo et al., 2020). During the poultry growth cycle, progressive bedding decomposition, along with the accumulation of animal residues, may create favorable conditions for fungal proliferation (Gomes et al., 2022).

*Aspergillus* section *Fumigati* species were frequently detected in bed and air samples, indicating that animal bedding material may represent an important matrix for fungal growth, as suggested before (Gomes et al., 2025a, 2022; Moreira et al., 2015; Viegas et al., 2012). Furthermore, indoor activities may contribute to the aerosolization and dissemination of small conidia, increasing their airborne abundance (Arné et al., 2021; Castro-Ríos et al., 2025; Sueiro-Olivares et al., 2015; Sugui et al., 2015).

As one of the most clinically relevant fungal pathogens, *Aspergillus fumigatus* can cause a wide spectrum of diseases (e.g., aspergillosis) in both humans and animals (Melo et al., 2020; Naeem et al., 2023). It is considered the leading pathogen within section *Fumigati* when compared with its closely related species (cryptic species), which generally exhibit lower pathogenic potential, mainly attributed to reduced thermotolerance (Lamoth, 2016; Melo et al., 2020). Although accurate species discrimination requires molecular analysis (e.g., internal transcribed spacer (ITS), ß-tubulin, or calmodulin sequencing) (Gonçalves et al., 2020; Lamoth, 2016), these analyses were not conducted in our study, representing a limitation. Nevertheless, our selection criteria (inoculation at 50 °C) were supported by previous evidence showing that fungal growth at elevated temperatures (> 48 °C) is indicative of *A. fumigatus sensu stricto* (Arendrup et al., 2024; Frías-De-León et al., 2023; Lamoth, 2016; Lestrade et al., 2019; Sugui et al., 2015).

Thermotolerance (at 37 °C) was used as a proxy for pathogenic potential, reflecting the fungal ability to colonize and develop at human body temperature and higher (Korfanty et al., 2023; Sugui et al., 2015). This is an advantage over body defense mechanisms, such as fever, and represents a key factor for the success of opportunistic fungi (Hernández-Benítez et al., 2025). Biofilm formation is another adaptive feature of this species, contributing to fungal virulence and resistance to several factors, including antifungal agents (Hernández-Benítez et al., 2025). Although these mechanisms were not evaluated in the present study, they may contribute to the recurrent detection of section *Fumigati* species throughout the poultry growth cycle, even in the empty pavilion (after cleaning and disinfection).

To more accurately estimate exposure risks, culture-based methods remain the gold standard, as they allow for assessing fungal viability and, consequently, the capacity of fungi to induce infection and trigger adverse effects within the host (Gomes et al., 2025a; Viegas et al., 2020b). In addition, these methods enable the recovery of isolates for further analysis, including the evaluation of cytotoxicity potential and antifungal resistance screening (Gomes et al., 2025a).

Considering *A.* section *Fumigati* prevalence, culture at higher temperatures (37 °C) yields the greatest results, which is consistent with the thermotolerant profile of this section (Frías-De-León et al., 2023; Korfanty et al., 2023; Sugui et al., 2015). These findings are particularly relevant for exposure assessment studies, as incubation at lower temperatures may promote the development of faster-growing fungi, potentially underestimating the presence of potentially pathogenic *Aspergillus* spp (Boff et al., 2012). Thus, our findings reinforce that exposure assessments targeting *A*. section *Fumigati* should include culture at 37 °C, as it seems to be the most appropriate temperature to recover section *Fumigati* isolates, enabling further analysis (Boff et al., 2012; Melo et al., 2020; Sugui et al., 2015).

Resistance to azoles, the antifungals of choice for prophylaxis and treatment of aspergillosis both in humans and animals, is a growing concern (Bottery et al., 2024; Melo et al., 2020), challenging the clinical management of fungal diseases (Dladla et al., 2024; Melo et al., 2020; Viegas et al., 2020b). This increase in resistance has been linked to the widespread use of azoles in agriculture and industrial applications (Celia-Sanchez et al., 2024; Dladla et al., 2024; Gonçalves et al., 2020), as recently highlighted by a joint report from five EU health and environment agencies (EFSA, ECDC, ECHA, EEA, EMA) (EFSA, 2025).

The emergence of ARAf has reinforced the need for standardized and reproducible antifungal susceptibility testing (AFST), both to guide clinical treatment and to monitor emerging resistance (Guinea et al., 2022). In this study, azole susceptibility testing was performed following the EUCAST reference broth microdilution method (E.Def 9.4, 2022), allowing the characterization of isolates according to their antifungal susceptibility profile (Serrano-Lobo et al., 2024). All seven isolates were classified as pan-azole-resistant. While concerning, these findings align with projections suggesting that resistance prevalence will continue to rise as a consequence of persistent environmental exposure to triazoles (Gonçalves et al., 2020; Lestrade et al., 2019).

Molecular studies have reported shared resistance mutations between environmental and clinical isolates (Snelders et al., 2009), supporting the hypothesis that resistant strains can easily transfer between these two domains (Dladla et al., 2024). Notably, three pan-azole-resistant isolates lacked the classical azole-resistance mutations. Instead, they exhibited eight naturally occurring polymorphisms (F46Y, M172V, N284T, D255E, E427K, C454C, L358L, G89G), along with indels in the *cyp51A* promoter region (up to 5 kb upstream). While the functional impact of these SNPs remains unclear, alterations in the *cyp51A* promoter have been associated with changes in gene expression and reduced azole susceptibility in *A.fumigatus* (Dladla et al., 2024; Hernández-Benítez et al., 2025). Thus, these distal promoter indels may represent candidate genetic features potentially contributing to the observed resistant phenotype. However, further studies, including gene expression analysis and functional validation, are required to determine their role in azole resistance.

The most commonly described mechanism of azole resistance involves tandem repeat (TR) insertions in the *cyp51A* gene promoter, in combination with single or multiple-point mutations (e.g., TR34/L98H, TR53, TR46/Y121F/T289A) (Celia-Sanchez et al., 2024; Dladla et al., 2024; Gonçalves et al., 2020). These genetic alterations modify the azole target enzyme, reducing antifungal binding affinity and thereby conferring resistance (Bottery et al., 2024; Dladla et al., 2024).

The environmental use of azole fungicides has been associated with the emergence and dissemination of TR-based resistance mechanisms in *A. fumigatus*, contributing to the global spread of resistant lineages, characterized by multi-azole resistance and increased recombination rates (Bottery et al., 2024; Celia-Sanchez et al., 2024). The four TR34/L98H pan-azole-resistant isolates identified in this study are consistent with the genetic profile described for this resistance lineage. This clade has been reported to frequently harbor the hypermutator variant msh6-G233A, which has been linked to increased mutation rates and adaptive capacity, potentially facilitating the evolution of antifungal resistance (Bottery et al., 2024). However, as the presence of the *msh6*-G233A variant was not assessed in the recovered isolates, its occurrence cannot be established. Nonetheless, the detection of TR34/L98H isolates in poultry farm environments highlights the importance of continued One Health surveillance to monitor the occurrence and potential dissemination of azole-resistant *A. fumigatus* across environmental, animal, and human interfaces.

Globally, environmental resistant strains have been mostly reported from agricultural environments, soil compost and plant debris (Arendrup et al., 2024; Bottery et al., 2024; Gómez Londoño and Brewer, 2023; Hoda et al., 2019; Prigitano et al., 2019). Some have been found in animal species, including zoo and companion animals, wildlife, horses and poultry, or in their feed (poultry) (Dieste-Pérez et al., 2025; Naeem et al., 2023). In Portugal, environmental strains of ARAf have only been identified by one study in dairy air and FRPD from waste sorting workers (Gonçalves et al., 2020). To the best of our knowledge, this is the first study reporting putative pan-azole-resistant *Aspergillus fumigatus* harboring the TR34/L98H mutation in environmental samples from poultry farms.

Our findings suggest that azole-resistant *A. fumigatus* occurs in poultry farm environments, with animal bedding representing an important matrix in which resistant isolates may occur, as previously suggested (Gomes et al., 2025a; 2022). The use of azole-treated wood shavings has been proposed as a potential source of ARAf in agricultural environments (EFSA, 2025), suggesting that bedding material may contribute to the introduction or maintenance of resistant fungal strains within farms (Gomes et al., 2025a, 2022; Gonçalves et al., 2020). However, azole residues in bedding material were not assessed in the present study, and the contribution of bedding treatment practices to the selection of resistant isolates within poultry farms cannot be confirmed. Nonetheless, the detection of resistant strains in an air sample emphasizes the increased exposure risk for both workers and animals (Gonçalves et al., 2020).

Toxicity studies allow the evaluation of harmful effects arising from complex mixtures or from fungal isolates (Gniadek et al., 2017; Gomes et al., 2025b). Exposure to *Aspergillus* section *Fumigati* and their mycotoxins has been associated with respiratory and hepatic disorders (Gniadek et al., 2017; Viegas et al., 2021b), which justifies the cell lines selected for this study, representing the respiratory organ (human lung epithelial [A549]) and detoxification organs (swine kidney [SK]; hepatic cells [HepG2]). The cytotoxicity results obtained for the isolates corroborate the known toxic potential of this species (Viegas et al., 2021a). While these results are essential to evaluate the potential impact of individual fungal species, the evaluation of the toxic effects of their corresponding environmental samples, previously described (Gomes et al., 2025b), more accurately reflects the real exposure scenario in poultry farms, where workers and animals are simultaneously exposed to biological and chemical agents (Dias et al., 2024; Gomes et al., 2025b).

The low agreement observed between environmental samples and fungal isolates suggests that culturable *A.* section *Fumigati* isolates alone are unlikely to explain the cytotoxicity detected in poultry farm environments. This finding indicates that other biological or chemical agents, including mycotoxins detected in these matrices, as well as interactions among different contaminants, may contribute to the overall toxicological profile of environmental samples. However, the present study does not allow the attribution of these metabolites to *A. fumigatus*, as they may originate from other fungal genera or contaminated environmental substrates.

Additional GEE analyses accounting for clustering within poultry farms showed no significant associations between cytotoxicity in fungal isolates and matrix, season, or production stage. In contrast, cytotoxicity in environmental samples was significantly influenced by matrix type and production stage, highlighting the importance of environmental conditions in shaping toxicological outcomes.

Considering the previously reported widespread occurrence of mycotoxins, including DON, FBs, and ZEA, in EDC, feed, and bed samples from poultry farms, and their well-established toxicological effects (Keutchatang et al., 2024; Kolawole et al., 2024), we further explored their potential contribution to the overall cytotoxicity observed. The association between mycotoxin detection and cytotoxicity depended on the selected cell line.

Regarding respiratory cells (A549), no significant association was observed in either the bivariate or adjusted analyses, suggesting that factors such as matrix type and production stage may have a greater influence on cytotoxicity than mycotoxin detection alone. In contrast, the association remained significant for SK cells, suggesting that mycotoxin detection was associated with increased odds of cytotoxicity in this cell line. This difference may reflect the distinct biological characteristics and susceptibility of the selected cell models (Viegas et al., 2022b), as mycotoxins can induce diverse cellular responses, including oxidative stress, inflammation, and metabolic disturbances, depending on the specific compound, exposure level, and target cell type (Keutchatang et al., 2024; Kolawole et al., 2024). However, these findings do not establish causality, and the contribution of mycotoxins or potential interactions among contaminants requires further investigation.

Collectively, these findings reinforce the need for continuous surveillance strategies targeting both *Aspergillus* spp. and mycotoxins in poultry production environments. Such initiatives should extend to poultry residues, including bedding material, and promote the development of standardized procedures for their management and safe land application. This approach aligns with the framework proposed by the recent Directive (EU) 2025/2360—Soil Monitoring Law, which emphasizes the assessment and monitoring of soil measures to improve soil health. Additionally, research efforts should focus on exploring sustainable antifungal alternatives, including plant essential oils, which have shown promising antifungal activity (Naeem et al., 2023; Tan et al., 2022). Working on these alternatives may contribute to reducing reliance on azoles and help mitigate the selective pressure associated with the emergence of azole resistance.

## 5 Conclusion

This study provides new evidence on the occurrence of putative azole-resistant *Aspergillus fumigatus* in poultry farms, with resistant isolates detected in both air and bedding material, thereby highlighting these matrices as important components of the poultry farm environment for future surveillance and exposure assessment.

In addition, the widespread occurrence of *Aspergillus* section *Fumigati*, together with the detection of mycotoxins and the observed cytotoxic responses in environmental samples, highlights the complexity of fungal exposure risks in poultry farms. Notably, this is the first report of putative pan-azole-resistant *A. fumigatus* carrying the TR34/L98H mutation in poultry farms.

Overall, these findings support the need for integrated surveillance strategies targeting both *Aspergillus* spp. and mycotoxins in poultry farms and reinforce the need for further research into azole resistance mechanisms and sustainable alternative compounds that reduce reliance on azole fungicides.

## Data Availability

The original contributions presented in the study are publicly available. This data can be found here: NCBI SRA, accession PRJNA1494181.
