# Supplementary material for: Detection of TR34/L98H pan-azole-resistant Aspergillus fumigatus in poultry farm environments
Source: Front Fungal Biol. 2026 Aug 19;7:1858050. doi: 10.3389/ffunb.2026.1858050 (PMC13534077; doi:10.3389/ffunb.2026.1858050)
Supplement: Supplementary file 1 [file DataSheet1.pdf]

Supplementary Material

Table S1 – Detection and concentration ranges (ng/g) of screened mycotoxins in environmental matrices (EDC, feed, bed) from poultry farms.

	EDC	FEED	BED
**Mycotoxins**	**Detection / Quantification range (ng/g)***	**Detection / Quantification range (ng/g)***	**Detection / Quantification range (ng/g)***
Aflatoxin B1(AFB1)	ND	ND	ND
Aflatoxin B2 (AFB2)	ND	ND	ND
Aflatoxin G1 (AFG1)	ND	ND	ND
Aflatoxin G2 (AFG2)	ND	ND	ND
Aflatoxin M1 (AFM1)	ND	ND	ND
Deoxynivalenol (DON)	Detected / [<LOQ (27) – 38.8]	Detected / [109 – 2890]	Detected / [<LOQ (26) – 97.1]
3-Acetyl-DON	ND	ND	ND
15-Acetyl-DON	ND	ND	ND
Nivalenol (NIV)	ND	Detected / [56.8]	ND
Fusarenon-X	ND	-	ND
T-2 toxin (T-2)	ND	Detected / [<LOQ (5.1) – 19.7]	ND
HT-2 toxin (HT-2)	ND	Detected / [<LOQ (12) – 32.5]	ND
Diacetoxyscirpenol (DAS)	ND	Detected / [<LOQ (8.6)]	ND
DON-3-glucoside	ND	Detected / [<LOQ (26) – 143]	ND
Deepoxy- deovynivalenol (DOM-1)	ND	ND	ND
Neosolaniol	ND	ND	ND
Monoacetoxyscirpenol	ND	ND	ND
T-2 tetraol	ND	ND	ND
T-2 triol	ND	ND	ND
Fumonisin B1(FB1)	Detected / [<LOQ (40.8) – 516]	Detected / [<LOQ (44) – 814]	Detected / [<LOQ (46) – 326]
Fumonisin B2 (FB2)	Detected / [<LOQ (25) – 118]	Detected / [<LOQ (36) – 405]	Detected / [<LOQ (38) – 92]
Fumonisin B3 (FB3)	ND	Detected / [<LOQ (8.6) – 212]	ND
Zearalenone (ZEN)	Detected / [<LOQ (4) – 4.09]	Detected / [<LOQ (2.6) – 400]	Detected / [<LOQ (3.2)– 6.62]
α-Zearalenol	ND	ND	ND
β-Zearalenol	ND	ND	ND
α-Zearalanol	ND	ND	ND
β-Zearalanol	ND	ND	ND
Zearalanone	ND	ND	ND
Ochratoxin A (OTA- A)	ND	Detected / [<LOQ (3.6)]	-ND
Ochratoxin B (OTA- B)	ND	ND	ND
Gliotoxin	ND	ND	ND
Griseofulvin	ND	ND	ND
Mycophenolic acid(MPA)	ND	ND	Detected / [<LOQ (4.9) – 888]
Mevinolin	ND	ND	-
Roquefortine C (ROQ -C)	ND	Detected / [<LOQ (12) – 0.90]	Detected / [<LOQ (12.2) – 872]
Sterigmatocystin (STER)	Detected / [<LOQ (4)]	ND	Detected / [<LOQ (1.2) – 3.38]
Patulin	ND	ND	ND
Moniliformin (MON)	ND	Detected / [<LOQ (28) – 315]	ND

**Limits of quantification (LOQ) are matrix-specific and provided in the table.*
ND – Not detected

Table S2- Azole resistance assessment

Section	Analysis	Procedure
2.5.2	Azole resistance screening	Putative *Aspergillus fumigatus* isolates were screened for azole resistance through the azole-containing agar plate method (EUCAST E.Def 10.1) following (Guinea et al. 2019). Inoculum suspensions followed the adaptation proposed by (Serrano-Lobo et al. 2024), in which suspensions are neither filtered nor adjusted. as these steps do not compromise screening results (Serrano-Lobo et al. 2024). Briefly, conidia suspension (25 uL) was inoculated into four-well RPMI-1640 agar plates (Sigma-Aldrich), where one well served as a growth control (antifungal-free well) and three wells were supplemented with 4 mg/L itraconazole (ITR). 2 mg/L voriconazole (VOR). and 0.5 mg/L posaconazole (POS), respectively. Plates were incubated at 37 °C, and the visual grow as assessed after 48 h. *Aspergillus fumigatus* isolates exhibiting growth on any of the azole-supplemented wells were considered potentially resistant and were further analysed.
2.5.3	Broth microdilutions susceptibility testing	Putative azole-resistant *Aspergillus fumigatus* (were tested for the antifungals itraconazole (ITR), voriconazole (VOR) and posaconazole (POS) respectively (Sigma. Madrid. Spain) at concentration ranging from 8 to 0.016 g/mL. Following the reference microdilution method (EUCAST E.Def 9.4) as described by (Guinea et al. 2022).The different concentrations were used to establish the minimum inhibitory concentrations (MICs), which is defined as the lowest antifungal concentration (mg/L) that inhibits fungal growth. Initially, isolates were subculture on potato dextrose agar and incubated at 35 ºC (2-5 days). Conidial suspensions were prepared in distilled water (0.1% Tween 20), serially diluted, quantified using a haemocytometer, and adjusted to obtain a final inoculum of 1-2.5 × 10^5 CFU/mL prior to inoculation onto microdilution plates. The plates were then incubated at 37 ºC for 48 h. Visual MIC readings were facilitated using a mirror and a black/white paper behind the microdilution plate. *Aspergillus flavus* ATCC 2004304 and *Aspergillus fumigatus* ATCC 2004305 served as quality control strains to verify assay performance. Isolates were classified according to EUCAST breakpoints (version 12.0. 2025). and were considered resistant when MIC breaking point >1 mg/L for itraconazole and voriconazole and MICs >0.125 mg/L for posaconazole (EUCAST, 2025).

Table S3- Cytotoxicity assessment

Section	Analysis	Procedure
2.6	Cytotoxicity evaluation of isolates	The assay was performed in A549 - human alveolar lung carcinoma cell line (ATCC® CCL-185™) and HepG2 - human hepatocellular carcinoma cell line (ATCC HB-8065) employing the 3-(4,5-dimethylthiazol-2-yl)-2,5-diphenyltetrazolium bromide (MTT) assay based on previously developed protocols (Gaspar et al., 2016). Cells were seeded in 96-well tissue culture plates at a density of 1×10^4 cells per well using RPMI 1640 culture medium (Gibco Life Technologies, Carlsbad, CA, USA) supplemented with 10% fetal bovine serum. 100 units/mL penicillin G (sodium salt). 100 µg/mL streptomycin sulfate, and 2 mM L-glutamine. The plates were incubated in a humidified atmosphere at 37 °C with 5% CO_2 for 24h. Subsequently, 10 µL of fungal suspension, prepared from pure colonies and adjusted to 0.5 McFarland standard, was added to each well containing pre-seeded cells. In negative control cell were incubated with culture medium and, in positive control sodium dodecyl sulfate (SDS) (0.1 mg/mL, Sigma) was added to promote cell lyses. . After 24 hours of incubation, the media was replaced with fresh medium containing 0.5 mg/mL of MTT dye. Following an additional 3-hour incubation, the media were removed, and the resulting intracellular formazan crystals were dissolved in dimethyl sulfoxide. The absorbance was then measured at 570 nm using a FLUOstar Omega Microplate Reader (BMG Labtech GmbH, Ortenberg. Germany) after 15 minutes stirring at room temperature. The relative cell viability (%) compared to control cells was calculated for the MTT assay using the following equation (1):<br><br>$(1)\ \text{Cell viability (\% of sample)} = 〚Abs〛\_sample / 〚Abs〛\_control \times 100$<br><br>where Abs sample is the absorbance value obtained for cells exposed to each isolate and Abs control is the absorbance value obtained for cells incubated with culture medium. Cytotoxicity was determined by analyzing cellular metabolic activity, which is a sensitive indicator of early-stage damage. The reduction of cell viability below 70 % was defined as the threshold at which a cytotoxic effect is considered to occur (Gaspar et al. 2016).

Table S4- Detection rates of *Aspergillus* section *Fumigati* by production stage at 27 ºC. 37 ºC. Results of the Chi-square test.Fisher-Freeman-Halton's. McNemar Test and effect size.

				Detection		Test Statistics			Effect size
				No	Yes	Qui-Square	df	p	
At 27ºC	Stage	0	n	44	8	3.986	3	0.263[a]	0.10[d]
			%	84.6	15.4				
		1	n	103	15				
			%	87.3	12.7				
		2	n	105	13				
			%	89.0	11.0				
		3	n	94	23				
			%	80.3	19.7				
	Total		n	346	59				
			%	85.40	14.60				
At 37ºC	Stage	0	n	1	51	1.969	3	0.699[b]	0.07[d]
			%	1.90	98.10				
		1	n	7	111				
			%	5.90	94.10				
		2	n	4	114				
			%	3.40	96.60				
		3	n	4	113				
			%	3.40	96.60				
	Total		n	16	389				
			%	4.00	96.00				

			Detection at 37ºC		Test Statistics			
			No	Yes				
			No	Yes	Qui-Square	df	p	Effect size
Detection at 27ºC	No	n	10	336	318.42	1	<0.001[c,*]	0,37[e]
		%	62.5	86.38				
	Yes	n	6	53				
		%	37.5	13.62				
	Total		16	389				

[a] Qui-Square Test. [b] Fisher-Freeman-Halton´s; [c]McNemar; Test. d. Cramer's V. e. Cohen's g

** Significant association at the 5% significance level*

Table S5- Azole-resistance screening of putative *Aspergillus fumigatus* isolates on itraconazole (ITR), voriconazole (VOR), posaconazole (POS).

		Azole -Susceptible(S) / - Potentially resistant (R)			
**MATRICE**	**Isolates collected (N)**	**ITR (4mg/L)**	**VOR (2 mg/L)**	**POS (0.5 mg/L)**	**% Resistance (R/N)**
AIR	41	41 S	40S/ 1R	40S/ 1R	2.44 % (1/41)
EDC	9	9 S	9 S	9 S	-
BED	41	36S/ 5R	35S/ 6R	35S/ 6R	14.63 % (6/41)
SWABS	40	40 S	40 S	40 S	-
FEED	27	27 S	27 S	27 S	-
FRPD	1	1 S	1 S	1 S	-
BREAST	5	5 S	5 S	5 S	-

Across the seven nanopore sequencing libraries, total output comprised 13.02 million reads and 48.76 Gb of sequence data. Per-sample yield ranged from 1.31 to 2.42 million reads and from 4.96 to 9.22 Gb. Mean read length varied from 2.94 to 4.50 kb. Mean read quality scores were closely distributed across samples, ranging from 13.1 to 14.7. The proportion of reads retained at Q10 was highly consistent, spanning 92.8–93.5% of reads and corresponding to 4.73–8.79 Gb per sample. At Q12, 76.6–78.8% of reads were retained, yielding 4.17–7.76 Gb per sample. At Q15, retention decreased to 40.7–46.6% of reads, corresponding to 2.86–5.39 Gb per sample. Kraken profiles were dominated by fungal assignments. At species level, reads were predominantly assigned to *Aspergillus fumigatus* across all datasets (ranging between 94.7% and 96.2% of the raw dataset). The principal non *A. fumigatus* species-level assignments were to other *Aspergillus* taxa, likely reflecting taxonomic ambiguity among closely related *Aspergillus* species. Unclassified reads were consistently low across raw and subsampled datasets, remaining below 1% (Table S6).

Table S6 - Summary of nanopore whole-genome sequencing quality metrics for the seven putative azole-resistant *Aspergillus fumigatus* isolates.

Sample	Reads	Bases	mean read len	mean qual	Reads Q10	Reads Q12	Reads Q15
157	$1x10^6$	6141528974	4271	13.1	1336895 (93.0%) 5868.7Mb	1119067 (77.8%) 5206.7Mb	638673 (44.4%) 3651.3Mb
160	$2x10^6$	7109057515	2942	14.2	2241679 (92.8%) 6773.7Mb	1851153 (76.6%) 5968.1Mb	985694 (40.8%) 4072.6Mb
226	$2x10^6$	4984378876	2971	14.3	1556768 (92.8%) 4740.1Mb	1293556 (77.1%) 4174.0Mb	682652 (40.7%) 2860.2Mb
229	$2x10^6$	7705737243	3683	14.5	1946554 (93.0%) 7335.9Mb	1628884 (77.8%) 6470.4Mb	910253 (43.5%) 4480.8Mb
245	$1x10^6$	4956415749	3789	14.4	1216831 (93.0%) 4725.4Mb	1018697 (77.9%) 4172.7Mb	566747 (43.3%) 2879.2Mb
268	$2x10^6$	8645249599	4237	14.6	1907575 (93.5%) 8253.2Mb	1605769 (78.7%) 7315.8Mb	915868 (44.9%) 5112.1Mb
269	$2x10^6$	9217996334	4499	14.7	1915942 (93.5%) 8790.9Mb	1615145 (78.8%) 7761.5Mb	954691 (46.6%) 5387.3Mb

Genome fraction was confined to a narrow interval across assemblies, spanning between 93% and 95%, with total aligned sequence ranging from 26.87 to 27.55 Mb. Assembly sizes ranged from 28.47 to 29.88 Mb, and contig counts varied, from 10 to 75 contigs. Total sequencing depth ranged from 168.7× to 313.7× relative to the 29.38 Mb *Aspergillus fumigatus* reference genome. Mapping revealed a constant primary read count of 1.214.013 across all samples and the proportion of reads classified as secondary or supplementary varied, resulting in total mapped percentages between 96% and 98% (Table S7).

Table S7 - Assembly and mapping statistics of the seven putative azole-resistant *Aspergillus fumigatus* genomes

Sample	Genome fraction (%)	Largest alignment	Total alignment length	NGA50	LGA50	Contigs	Largest contig	Total length	Depth (x)
157	94.572	1632646	27503725	254301	34	75	2904938	29661245	209
160	93.009	639202	26868975	185952	48	12	4832880	28468471	242
226	93.084	639203	26922719	185954	48	10	4832933	28526845	170
229	93.09	639208	26924712	185951	48	11	4832815	28493592	262
245	93.739	829888	27132101	235960	37	15	4819657	28840850	169
268	93.785	829897	27135435	235963	37	19	4807612	28689918	294
269	94.756	638513	27545562	189253	48	61	4001170	29876266	314

Table S8 - Cytotoxicity effects of *Aspergillus* section *Fumigati* isolates on human alveolar lung carcinoma cell line (A549) and human human hepatocellular carcinoma HepG2 cells. The results (%) of cell viability (CV). standard deviation (SD) and cytotoxicity: "1" : cytotoxic effect; "0": no cytotoxic effect)

Isolate	% CV A545	SD	A549 cytotoxic	% CV HepG2	SD	HepG2 cytotoxic
1	90	12	0	77	13	0
2	96	19	0	73	27	0
3	86	7	0	66	17	1
4	84	10	0	68	11	1
5	84	14	0	73	12	0
6	79	15	0	84	19	0
14	75	6	0	72	12	0
15	87	17	0	89	15	0
16	67	7	1	60	7	1
17	70	5	0	48	12	1
18	77	0	0	40	0	1
19	92	14	0	60	27	1
21	89	15	0	80	0	0
25	89	12	0	59	3	1
76	69	3	1	58	13	1
80	82	7	0	78	9	0
83	63	8	1	63	7	1
84	69	8	1	78	12	0
85	66	14	1	66	6	1
86	64	4	1	67	4	1
88	77	7	0	67	19	1
95	78	2	0	60	8	1
98	94	22	0	87	26	0
99	85	16	0	66	14	1
100	69	2	1	68	10	1
101	85	7	0	73	16	0
105	81	1	0	60	10	1
106	65	6	1	61	6	1
107	65	6	1	50	6	1
108	63	4	1	36	6	1
109	85	14	0	84	8	0

(Continued)

110	90	12	0	76	11	0
113	79	6	0	85	4	0
115	99	34	0	71	3	0
116	96	12	0	70	9	0
119	72	13	0	56	13	1
120	68	18	1	58	13	1
122	84	11	0	70	9	1
124	79	4	0	75	5	0
159	89	22	0	72	9	0
160	76	3	0	69	10	1
157	70	2	0	61	4	1
164	79	12	0	66	10	1
165	70	1	0	65	11	1
167	76	8	0	68	7	1
168	79	3	0	78	23	0
169	76	4	0	69	16	1
170	93	10	0	69	4	1
171	88	6	0	66	11	1
173	73	13	0	63	7	1
172	69	1	1	83	1	0
174	73	9	0	68	14	1
175	69	43	1	72	5	0
176	68	11	1	56	27	1
177	75	6	0	70	5	0
178	81	18	0	61	3	1
179	66	5	1	56	8	1
180	79	12	0	67	10	1
222	69	5	1	65	8	1
223	80	17	0	65	11	1
225	95	14	0	83	23	0
226	83	9	0	84	16	0
227	70	8	0	80	15	0
228	71	3	0	65	4	1
229	74	9	0	71	14	0
230	84	14	0	77	6	0
231	82	2	0	85	15	0

(Continued)

234	71	3	0	65	11	1
235	79	15	0	69	11	1
236	76	4	0	91	11	0
238	82	15	0	86	5	0
239	72	4	0	72	5	0
240	75	6	0	63	6	1
241	58	10	1	34	8	1
243	71	4	0	73	23	0
245	67	9	1	54	9	1
246	69	5	1	64	9	1
247	67	1	1	66	7	1
251	88	4	0	98	0	0
255	90	0	0	69	10	1
261	91	9	0	95	0	0
263	79	17	0	58	9	1
265	97	1	0	87	8	0
268	94	0	0	89	15	0
269	90	19	0	67	42	1
270	85	13	0	86	7	0
271	95	14	0	85	5	0
272	84	15	0	52	6	1
273	98	7	0	99	0	0
298	95	4	0	86	0	0
299	93	0	0	85	14	0
300	57	10	1	91	21	0
301	85	15	0	67	9	1
302	78	16	0	65	14	1
330	80	9	0	74	14	0
335	82	6	0	80	12	0
349	87	7	0	69	2	1
353	76	18	0	64	15	1
356	100	0	0	74	9	0
357	97	9	0	92	11	0
359	91	12	0	94	19	0
366	78	15	0	90	39	0

(Continued)

371	100	25	0	85	21	0
374	84	13	0	52	27	1
375	86	14	0	87	12	0
489	89	9	0	73	5	0
494	89	9	0	94	7	0
508	80	14	0	77	11	0
573	93	9	0	76	36	0
581	90	2	0	79	29	0
648	97	0	0	87	15	0
672	92	2	0	79	17	0
675	72	16	0	55	8	1
737	95	21	0	57	29	1
740	85	17	0	66	7	1
741	87	15	0	46	12	1
744	94	10	0	67	16	1
747	83	10	0	48	14	1
748	92	15	0	59	29	1
836	96	14	0	82	14	0
839	93	2	0	76	19	0
844	61	12	1	75	20	0
847	77	21	0	61	15	1
850	95	22	0	86	19	0
853	95	17	0	35	2	1
855	89	10	0	71	17	0
856	98	4	0	68	20	1
857	78	9	0	70	14	0
862	88	4	0	72	39	0
865	86	11	0	95	12	0
867	91	18	0	91	15	0
894	61	12	1	90	5	0
902	80	17	0	82	8	0
922	74	23	0	90	7	0
925	96	6	0	77	8	0
934	88	12	0	77	7	0

(Continued)

937	79	10	0	67	21	1
954	87	11	0	70	21	1
938	85	13	0	88	2	0
941	86	13	0	82	20	0
942	87	2	0	81	12	0
950	94	8	0	72	5	0
952	73	22	0	65	15	1
963	67	13	1	91	9	0

(Continued)

Table S9 - Cytotoxicity results of fungal isolates according to sample matrix. Values are presented as the number of cytotoxic isolates/total isolates (n/N) and percentage (%). Cytotoxicity was evaluated in A549 and HepG2 cell lines and classified as a binary outcome (0 = non-cytotoxic; 1 = cytotoxic).

	**HepG2 cytotoxic**		**A549 cytotoxic**	
**Matrix**	**n/N**	**%**	**n/N**	**%**
AIR	25/38	65.79	7/38	18.42
EDC	0/7	0.00	0/7	0.00
FEED	11/25	44.00	2/25	8.00
BED	16/34	47.06	5/34	14.71
SWABS	14/35	40.00	9/35	25.71
FRPD	0/1	0.00	1/1	100.00
BROILERS BREAST	1/5	20.00	0/5	0.00
**TOTAL**	**67/145**	**46.21**	**24/145**	**16.55**

Table S10 – Comparison of cytotoxicity results ("0":no cytotoxic effects; "1": cytotoxic) in respiratory cells line (A549 - human alveolar lung carcinoma) from fungal isolates and their corresponding environmental samples

						Cytotoxicity results (A549 cells)	
Isolate ID	Sample ID	Farm	Stage	Matrice	SEASON	Isolate	Sample
222	BB_1	B	1	BED	WINTER	1	1
223	BC_1	C	1	BED	WINTER	0	1
225	BD_1	D	1	BED	WINTER	0	1
226	BA_2	A	2	BED	WINTER	0	1
227	BA_1	A	1	BED	WINTER	0	1
228	BB_2	B	2	BED	WINTER	0	1
229	AB_2	B	2	BED	WINTER	0	1
230	AC_2	C	2	BED	WINTER	0	1
231	AD_2	D	2	BED	WINTER	0	1
232	C_0	A	0	BED	WINTER	0	1
234	AB_3	B	3	BED	WINTER	0	0
235	AC_3	C	3	BED	WINTER	0	1
236	EB_3	B	3	BED	WINTER	0	1
238	AD_1	D	1	BED	WINTER	0	1
239	EA_3	A	3	BED	WINTER	0	1
240	EB_2	B	2	BED	WINTER	0	1
241	BD_2	D	2	BED	WINTER	1	1
243	BC_2	C	2	BED	WINTER	0	1
245	AB_1	B	1	BED	WINTER	1	1
246	CB_3	B	3	BED	WINTER	1	1
247	CB_2	B	2	BED	WINTER	1	1
251	CA_3	A	3	BED	WINTER	0	1
255	CB_1	B	1	BED	WINTER	0	1
261	EB_1	B	1	BED	WINTER	0	1
263	E_0	A	0	BED	WINTER	0	0
265	DA_1	A	1	BED	WINTER	0	1
268	BD_3	D	3	BED	WINTER	0	1
269	AA_3	A	3	BED	WINTER	0	0
270	AA_1	A	1	BED	WINTER	0	1

**Sample IDs contain information regarding the sampling origin. For example, BB_1 indicates farm B (first letter), poultry pavilion B (second letter), and production stage 1 (number).*
(Continued)

272	BA_3	A	3	BED	WINTER	0	1
273	BB_3	B	3	BED	WINTER	0	1
298	AB_2	B	2	FEED	WINTER	0	1
299	AD_1	D	1	FEED	WINTER	0	1
300	BC_3	C	3	FEED	WINTER	1	1
301	B_0	A	0	FEED	WINTER	0	1
302	AC_1	C	1	FEED	WINTER	0	0
330	EB_1	B	1	EDC	WINTER	0	0
353	EA_2	A	2	FEED	WINTER	0	0
356	CB_3	B	3	FEED	WINTER	0	1
357	DB_2	B	2	FEED	WINTER	0	1
359	EA_1	A	1	FEED	WINTER	0	1
366	D_1	A	0	FEED	WINTER	0	0
371	DA_1	A	1	FEED	WINTER	0	0
374	CB_1	B	1	FEED	WINTER	0	1
375	EA_1	A	1	EDC	WINTER	0	0
489	CA_0	A	0	EDC	WINTER	0	0
494	CB_3	B	3	EDC	WINTER	0	0
508	2DB_2	B	2	EDC	SUMMER	0	0
573	2BA_3	A	3	EDC	SUMMER	0	1
581	2BC_2	C	2	EDC	SUMMER	0	1
648	2AD_2	D	2	BED	SUMMER	0	1
737	2D_0	A	0	FEED	SUMMER	0	1
740	2DB_1	B	1	FEED	SUMMER	0	1
741	2DA_2	A	2	FEED	SUMMER	0	1
744	2DB_3	B	3	FEED	SUMMER	0	0
836	2BB_3	B	3	FEED	SUMMER	0	1
839	2BC_3	C	3	FEED	SUMMER	0	1
844	2EA_1	A	1	FEED	SUMMER	1	0
847	2EA_2	A	2	FEED	SUMMER	0	1
850	2EB_2	B	2	FEED	SUMMER	0	0
853	2BA_2	A	2	FEED	SUMMER	0	1
855	2BB_2	B	2	FEED	SUMMER	0	1
856	2B_1	A	0	FEED	SUMMER	0	1
857	2B_2	A	0	FEED	SUMMER	0	1

**Sample IDs contain information regarding the sampling origin. For example, BB_1 indicates farm B (first letter), poultry pavilion B (second letter), and production stage 1 (number).*
(Continued)

Table S11 - Results of generalized estimating equations (GEE) with binomial distribution and logit link function for cytotoxicity induced by *Aspergillus* section *Fumigati* isolates in A549 cells.

Parameter	B	Std. Error	95% Wald Confidence Interval		Odds Ratio	95% OR Confidence Interval		Hypothesis Test		
			Lower	Upper		Lower	Upper	Wald Chi-Square	df	p
(Intercept)	-2.358	1.502	-5.301	0.586				2.464	1	0.116
Matrice=BED	0.722	1.303	-1.834	3.277	2.058	0.160	26.504	0.306	1	0.580
Matrice=EDC	-25.902[a]									
Matrice=FEED	0[b]									
Season=Summer	-0.297	1.233	-2.714	2.120	0.743	0.066	8.334	0.058	1	0.810
Season=Winter	0[b]									
Stage=0	-26.531[a]									
Stage=1	0.225	0.869	-1.478	1.927	1.252	0.228	6.871	0.067	1	0.796
Stage=2	-0.144	0.763	-1.639	1.351	0.866	0.194	3.861	0.036	1	0.850
Stage=3	0[b]									
(Scale)	1									

Dependent Variable: A545_Isolado
Model: (Intercept), Matrice, Season, Stage

a. Hessian matrix singularity is caused by this parameter. The parameter estimate at the last iteration is displayed.

b. Set to zero because this parameter is redundant.

Table S12- Results of generalized estimating equations (GEE) with binomial distribution and logit link function for cytotoxicity induced by environmental samples in A549 cells.

Parameter	B	Std. Error	95% Wald Confidence Interval		Odds Ratio	95% OR Confidence Interval		Hypothesis Test		
			Lower	Upper		Lower	Upper	Wald Chi-Square	df	p
(Intercept)	0.409	0.354	-0.284	1.103				1.338	1	0.247
Matrice=BED	1.927	0.423	1.098	2.756	6.868	2.997	15.739	20.736	1	0.000
Matrice=EDC	-2.010	0.731	-3.444	-0.577	0.134	0.032	0.561	7.558	1	0.006
Matrice=FEED	0[a]									
Season=Summer	0.657	0.939	-1.184	2.498	1.929	0.306	12.156	0.489	1	0.484
Season=Winter	0[a]									
Stage=0	-1.696	0.945	-3.548	0.155	0.183	0.029	1.168	3.225	1	0.073
Stage=1	0.064	0.697	-1.301	1.430	1.066	0.272	4.178	0.008	1	0.927
Stage=2	1.272	0.141	0.996	1.547	3.567	2.708	4.698	81.793	1	0.000
Stage=3	0[a]									
Scale	1									

Dependent Variable: A545_Amostra
Model: (Intercept), Matrice, Season, Stage

a. Set to zero because this parameter is redundant.

Table S13- Comparison of cytotoxicity results (“0”:no cytotoxic effects; “1”:cytotoxic) on human alveolar epithelial (A549). swine kidney (SK) cells and mycotoxin detection (“0”:no detected; “1”: detected) in environmental samples.

Sample ID	Farm	Stage	Matrix	Season	Cytotoxicity results		Mycotoxins detection
					SK cells	A549 cells	
A_I0	A	0	BED	WINTER	1	1	0
AA_1	A	1	BED	WINTER	1	1	1
AB_1	A	1	BED	WINTER	1	1	1
AC_1	A	1	BED	WINTER	1	1	1
AD_1	A	1	BED	WINTER	1	1	0
AA_2	A	2	BED	WINTER	1	1	1
AB_2	A	2	BED	WINTER	1	1	1
AC_2	A	2	BED	WINTER	1	1	1
AD_2	A	2	BED	WINTER	1	1	1
AA_3	A	3	BED	WINTER	1	0	1
AB_3	A	3	BED	WINTER	1	0	1
AC_3	A	3	BED	WINTER	1	1	1
AD_3	A	3	BED	WINTER	1	1	1
B_0	B	0	BED	WINTER	1	1	1
BA_1	B	1	BED	WINTER	1	1	1
BB_1	B	1	BED	WINTER	1	1	1
BC_1	B	1	BED	WINTER	1	1	1
BD_1	B	1	BED	WINTER	1	1	1
BA_2	B	2	BED	WINTER	1	1	1
BB_2	B	2	BED	WINTER	1	1	1
BC_2	B	2	BED	WINTER	1	1	1
BD_2	B	2	BED	WINTER	1	1	1
BA_3	B	3	BED	WINTER	1	1	1
BB_3	B	3	BED	WINTER	1	1	1
BC_3	B	3	BED	WINTER	1	1	1
BD_3	B	3	BED	WINTER	1	1	1
C_0	C	0	BED	WINTER	1	1	1
CA_1	C	1	BED	WINTER	1	1	1
CB_1	C	1	BED	WINTER	1	1	1
CA_2	C	2	BED	WINTER	1	1	1
CB_2	C	2	BED	WINTER	1	1	1
CA_3	C	3	BED	WINTER	1	1	0
CB_3	C	3	BED	WINTER	1	1	1
D_0	D	0	BED	WINTER	1	0	1
DA_1	D	1	BED	WINTER	1	1	1
DB_1	D	1	BED	WINTER	1	0	1
DA_2	D	2	BED	WINTER	1	1	0
DB_2	D	2	BED	WINTER	1	1	1
DA_3	D	3	BED	WINTER	1	1	1

**Sample IDs contain information regarding the sampling origin. For example, BB_1 indicates farm B (first letter), poultry pavilion B (second letter), and production stage 1 (number).*
(Continued)

Sample ID	Farm	Stage	Matrix	Season	Cytotoxicity results		Mycotoxins detection
					SK cells	A549 cells	
DB_3	D	3	BED	WINTER	1	1	1
E_0	E	0	BED	WINTER	1	0	0
EA_1	E	1	BED	WINTER	1	1	1
EB_1	E	1	BED	WINTER	1	1	1
EA_2	E	2	BED	WINTER	1	1	1
EB_2	E	2	BED	WINTER	1	1	1
EA_3	E	3	BED	WINTER	1	1	0
EB_3	E	0	BED	WINTER	1	1	0
AA_1	A	1	FEED	WINTER	1	0	1
AB_1	A	1	FEED	WINTER	1	1	1
AC_1	A	1	FEED	WINTER	1	0	1
AD_1	A	1	FEED	WINTER	1	1	1
AA_2	A	2	FEED	WINTER	1	1	1
AB_2	A	2	FEED	WINTER	1	1	1
AC_2	A	2	FEED	WINTER	1	1	1
AD_2	A	2	FEED	WINTER	1	1	1
AA_3	A	3	FEED	WINTER	1	1	1
AB_3	A	3	FEED	WINTER	0	1	1
AC_3	A	3	FEED	WINTER	1	0	1
AD_3	A	3	FEED	WINTER	0	1	1
BA_1	B	1	FEED	WINTER	1	0	1
BB_1	B	1	FEED	WINTER	1	1	1
BC_1	B	1	FEED	WINTER	1	0	1
BD_1	B	1	FEED	WINTER	1	0	1
BA_2	B	2	FEED	WINTER	1	0	1
BB_2	B	2	FEED	WINTER	0	1	1
BC_2	B	2	FEED	WINTER	1	0	1
BA_3	B	3	FEED	WINTER	1	0	1
BB_3	B	3	FEED	WINTER	1	1	1
BC_3	B	3	FEED	WINTER	1	0	1
CA_1	C	1	FEED	WINTER	0	1	1
CB_1	C	1	FEED	WINTER	1	1	1
CA_2	C	2	FEED	WINTER	0	1	1
CB_2	C	2	FEED	WINTER	1	0	1
CA_3	C	3	FEED	WINTER	0	1	1
CB_3	C	3	FEED	WINTER	1	1	1
DA_1	D	1	FEED	WINTER	0	0	1
DB_1	D	1	FEED	WINTER	0	0	1
DA_2	D	2	FEED	WINTER	0	0	1
DB_2	D	2	FEED	WINTER	0	1	1
DA_3	D	3	FEED	WINTER	1	1	1
DB_3	D	3	FEED	WINTER	1	1	1
EA_1	E	1	FEED	WINTER	1	1	1

**Sample IDs contain information regarding the sampling origin. For example, BB_1 indicates farm B (first letter), poultry pavilion B (second letter), and production stage 1 (number).*
(Continued)

Sample ID	Farm	Stage	Matrix	Season	Cytotoxicity results		Mycotoxins detection
					SK cells	A549 cells	
EB_1	E	1	FEED	WINTER	1	1	1
EA_2	E	2	FEED	WINTER	1	0	1
EB_2	E	2	FEED	WINTER	0	1	1
EA_3	E	3	FEED	WINTER	1	1	1
EB_3	E	3	FEED	WINTER	1	1	1
A_I0	A	0	EDC	WINTER	1	0	0
A_1	A	1	EDC	WINTER	1	0	0
AB_1	A	1	EDC	WINTER	1	0	0
AC_1	A	1	EDC	WINTER	1	0	0
AD__1	A	1	EDC	WINTER	1	0	0
AA__2	A	2	EDC	WINTER	1	0	0
AB_2	A	2	EDC	WINTER	1	0	0
AC_2	A	2	EDC	WINTER	1	0	0
AD_2	A	2	EDC	WINTER	1	0	0
AA_3	A	3	EDC	WINTER	1	0	0
AB_3	A	3	EDC	WINTER	1	0	0
AC_3	A	3	EDC	WINTER	1	0	0
AD_3	A	3	EDC	WINTER	1	0	1
B_0	B	0	EDC	WINTER	1	0	0
BA_1	B	1	EDC	WINTER	1	0	0
BB_1	B	1	EDC	WINTER	1	0	0
BC_1	B	1	EDC	WINTER	1	0	0
BD_1	B	1	EDC	WINTER	1	0	0
BA_2	B	2	EDC	WINTER	1	0	1
BB_2	B	2	EDC	WINTER	0	0	1
BC_2	B	2	EDC	WINTER	0	0	1
BD_2	B	2	EDC	WINTER	0	0	1
BA_3	B	3	EDC	WINTER	0	0	1
BB_3	B	3	EDC	WINTER	1	0	1
BC_3	B	3	EDC	WINTER	0	0	1
BD_3	B	3	EDC	WINTER	0	0	1
C_I0	C	0	EDC	WINTER	1	0	0

**Sample IDs contain information regarding the sampling origin. For example, BB_1 indicates farm B (first letter), poultry pavilion B (second letter), and production stage 1 (number).*
(Continued)

Sample ID	Farm	Stage	Matrix	Season	Cytotoxicity results		Mycotoxins detection
					SK cells	A549 cells	
CA_1	C	1	EDC	WINTER	1	0	1
CB_1	C	1	EDC	WINTER	1	0	1
CA_2	C	2	EDC	WINTER	0	0	1
CB_2	C	2	EDC	WINTER	0	0	1
CA_3	C	3	EDC	WINTER	0	0	1
CB_3	C	3	EDC	WINTER	0	0	1
D_I0	D	0	EDC	WINTER	0	0	0
DA_1	D	1	EDC	WINTER	1	0	0
DB_1	D	1	EDC	WINTER	0	0	0
DA_2	D	2	EDC	WINTER	0	0	1
DB_2	D	2	EDC	WINTER	0	0	1
DA_3	D	3	EDC	WINTER	0	0	1
DB_3	D	3	EDC	WINTER	0	0	1
E_0	E	0	EDC	WINTER	0	0	0
EA_1	E	1	EDC	WINTER	0	0	0
EB_1	E	1	EDC	WINTER	0	0	0
EA_2	E	2	EDC	WINTER	0	0	1
EB_2	E	2	EDC	WINTER	0	0	1
EA_3	E	3	EDC	WINTER	0	0	1
EB_3	E	3	EDC	WINTER	0	0	1
2A_0	A	0	BED	SUMMER	1	1	0
2AA_1	A	1	BED	SUMMER	0	1	0
2AB_1	A	1	BED	SUMMER	0	1	0
2AC_1	A	1	BED	SUMMER	1	1	0
2AD_1	A	1	BED	SUMMER	1	1	0
2AA_2	A	2	BED	SUMMER	0	1	0
2AB_2	A	2	BED	SUMMER	0	1	0
2AC_2	A	2	BED	SUMMER	0	1	0
2AD_2	A	2	BED	SUMMER	0	1	1
2AA_3	A	3	BED	SUMMER	0	1	1
2AB_3	A	3	BED	SUMMER	0	1	0
2AC_3	A	3	BED	SUMMER	0	1	0
2AD_3	A	3	BED	SUMMER	1	1	0

**Sample IDs contain information regarding the sampling origin. For example, BB_1 indicates farm B (first letter), poultry pavilion B (second letter), and production stage 1 (number).*
(Continued)

Sample ID	Farm	Stage	Matrix	Season	Cytotoxicity results		Mycotoxins detection
					SK cells	A549 cells	
2B_0	B	0	BED	SUMMER	1	1	0
2BA_1	B	1	BED	SUMMER	1	0	0
2BB_1	B	1	BED	SUMMER	0	0	0
2BC_1	B	1	BED	SUMMER	0	1	1
2BD_1	B	1	BED	SUMMER	0	0	0
2BA_2	B	2	BED	SUMMER	0	0	0
2BB_2	B	2	BED	SUMMER	0	1	0
2BC_2	B	2	BED	SUMMER	1	1	0
2BD_2	B	2	BED	SUMMER	1	0	0
2BA_3	B	3	BED	SUMMER	1	1	0
2BB_3	B	3	BED	SUMMER	1	1	0
2BC_3	B	3	BED	SUMMER	0	1	0
2BD_3	B	3	BED	SUMMER	0	1	0
2D_0	D	0	BED	SUMMER	0	1	0
2DA_1	D	1	BED	SUMMER	0	1	0
2DB_1	D	1	BED	SUMMER	0	1	0
2DA_2	D	2	BED	SUMMER	0	1	0
2DB_2	D	2	BED	SUMMER	0	1	0
2DA_3	D	3	BED	SUMMER	0	1	0
2DB_3	D	3	BED	SUMMER	1	1	0
2E_I0	E	0	BED	SUMMER	1	1	0
2EA_1	E	1	BED	SUMMER	0	1	0
2EB_1	E	1	BED	SUMMER	1	1	1
2EA_2	E	2	BED	SUMMER	1	1	0
2EB_2	E	2	BED	SUMMER	1	1	0
2EA_3	E	3	BED	SUMMER	1	1	0
2EB_3	E	3	BED	SUMMER	0	1	1
2AA_1	A	1	FEED	SUMMER	1	1	1
2AB_1	A	1	FEED	SUMMER	1	1	1
2AC_1	A	1	FEED	SUMMER	1	1	1
2AD_1	A	1	FEED	SUMMER	1	1	1
2AA_2	A	2	FEED	SUMMER	1	1	1
2AB_2	A	2	FEED	SUMMER	1	0	1
2AC_2	A	2	FEED	SUMMER	1	1	1
2AD_2	A	2	FEED	SUMMER	1	1	1
2AA_3	A	3	FEED	SUMMER	1	1	1
2AB_3	A	3	FEED	SUMMER	1	1	1
2AC_3	A	3	FEED	SUMMER	1	1	1
2AD_3	A	3	FEED	SUMMER	0	1	1

**Sample IDs contain information regarding the sampling origin. For example, BB_1 indicates farm B (first letter), poultry pavilion B (second letter), and production stage 1 (number).*
(Continued)

Sample ID	Farm	Stage	Matrix	Season	Cytotoxicity results SK cells	A549 cells	Mycotoxins detection
2BA_1	B	1	FEED	SUMMER	1	0	1
2BB_1	B	1	FEED	SUMMER	1	1	1
2BC_1	B	1	FEED	SUMMER	1	1	1
2BD_1	B	1	FEED	SUMMER	1	1	1
2BA_2	B	2	FEED	SUMMER	1	1	1
2BB_2	B	2	FEED	SUMMER	1	1	1
2BC_2	B	2	FEED	SUMMER	1	1	1
2BD_2	B	2	FEED	SUMMER	1	1	1
2BA_3	B	3	FEED	SUMMER	1	1	1
2BB_3	B	3	FEED	SUMMER	1	1	1
2BC_3	B	3	FEED	SUMMER	1	1	1
2BD_3	B	3	FEED	SUMMER	1	1	1
2DA_1	D	1	FEED	SUMMER	0	1	1
2DB_1	D	1	FEED	SUMMER	1	1	1
2DA_ 2	D	2	FEED	SUMMER	1	1	1
2DB_ 2	D	2	FEED	SUMMER	1	1	1
2DA_3	D	3	FEED	SUMMER	1	0	1
2DB_3	D	3	FEED	SUMMER	0	0	1
2EA_1	E	1	FEED	SUMMER	1	0	1
2EB_1	E	1	FEED	SUMMER	1	1	1
2EA_2	E	2	FEED	SUMMER	1	1	1
2EB_2	E	2	FEED	SUMMER	1	0	1
2EA_3	E	3	FEED	SUMMER	1	1	1
2A_0	A	0	EDC	SUMMER	0	1	0
2AA_1	A	1	EDC	SUMMER	0	1	0
2AB_1	A	1	EDC	SUMMER	0	1	0
2AC_1	A	1	EDC	SUMMER	1	0	0
2AD_1	A	1	EDC	SUMMER	0	0	0
2AA_2	A	2	EDC	SUMMER	0	1	0
2AB_2	A	2	EDC	SUMMER	0	1	0
2AC_2	A	2	EDC	SUMMER	0	0	0
2AD_2	A	2	EDC	SUMMER	1	1	0
2AA_3	A	3	EDC	SUMMER	1	1	0
2AB_3	A	3	EDC	SUMMER	1	1	0
2AC_3	A	3	EDC	SUMMER	0	1	0
2AD_3	A	3	EDC	SUMMER	0	1	0

**Sample IDs contain information regarding the sampling origin. For example, BB_1 indicates farm B (first letter), poultry pavilion B (second letter), and production stage 1 (number).*
(Continued)

Sample ID	Farm	Stage	Matrix	Season	Cytotoxicity results SK cells	A549 cells	Mycotoxins detection
2B_0	B	0	EDC	SUMMER	1	0	0
2BA_1	B	1	EDC	SUMMER	0	1	1
2AC_1	A	1	EDC	SUMMER	1	0	0
2AD_1	A	1	EDC	SUMMER	0	0	0
2AA_2	A	2	EDC	SUMMER	0	1	0
2AB_2	A	2	EDC	SUMMER	0	1	0
2AC_2	A	2	EDC	SUMMER	0	0	0
2AD_2	A	2	EDC	SUMMER	1	1	0
2AA_3	A	3	EDC	SUMMER	1	1	0
2AB_3	A	3	EDC	SUMMER	1	1	0
2AC_3	A	3	EDC	SUMMER	0	1	0
2AD_3	A	3	EDC	SUMMER	0	1	0
2B_0	B	0	EDC	SUMMER	1	0	0
2BA_1	B	1	EDC	SUMMER	0	1	1
2BB_1	B	1	EDC	SUMMER	1	1	1
2BC_1	B	1	EDC	SUMMER	0	1	0
2BD_1	B	1	EDC	SUMMER	1	1	0
2BA_2	B	2	EDC	SUMMER	1	0	1
2BB_2	B	2	EDC	SUMMER	0	1	1
2BC_2	B	2	EDC	SUMMER	1	1	1
2BD_2	B	2	EDC	SUMMER	1	1	1
2BA_3	B	3	EDC	SUMMER	1	1	1
2BB_3	B	3	EDC	SUMMER	1	1	1
2BC_3	B	3	EDC	SUMMER	1	1	1
2BD_3	B	3	EDC	SUMMER	0	1	1
2D_0	D	0	EDC	SUMMER	1	1	0
2DA_1	D	1	EDC	SUMMER	1	1	0
2DB_1	D	1	EDC	SUMMER	1	1	0
2DA_2	D	2	EDC	SUMMER	1	1	1
2DB_2	D	2	EDC	SUMMER	0	0	0
2DA_3	D	3	EDC	SUMMER	0	1	0
2DB_3	D	3	EDC	SUMMER	0	0	0
2E_0	E	0	EDC	SUMMER	1	1	0
2EA_1	E	1	EDC	SUMMER	1	0	0
2EB_1	E	1	EDC	SUMMER	0	1	0
2EA_2	E	2	EDC	SUMMER	0	1	0
2EB_2	E	2	EDC	SUMMER	1	0	0

**Sample IDs contain information regarding the sampling origin. For example, BB_1 indicates farm B (first letter), poultry pavilion B (second letter), and production stage 1 (number).*
(Continued)

Sample ID	Farm	Stage	Matrix	Season	SK cells	A549 cells	Mycotoxins detection
2EB_1	E	1	EDC	SUMMER	0	1	0
2EA_2	E	2	EDC	SUMMER	0	1	0
2EB_2	E	2	EDC	SUMMER	1	0	0
2EA_3	E	3	EDC	SUMMER	0	0	1
2EB_3	E	3	EDC	SUMMER	0	1	1

**Sample IDs contain information regarding the sampling origin. For example, BB_1 indicates farm B (first letter), poultry pavilion B (second letter), and production stage 1 (number).*
(Continued)

Table S14 – Cytotoxicity outcomes in SK and A549 cell lines and mycotoxin detection according to environmental matrices and production stages. Values are presented as number of positive samples/total samples (n/N) and percentage (%). Cytotoxicity and mycotoxin detection were analysed as binary outcomes (0 = no cytotoxic effect/not detected; 1 = cytotoxic effect/detected respectively).

		**SK cytotoxic (n/N; %)**		**A549 cytotoxic (n/N; %)**		**Mycotoxins detection (n/N; %)**	
**Matrix**	**Stage**	**n/N**	**%**	**n**	**%**	**n**	**%**
EDC	0	6/9	66.67	3/9	33.33	0/9	0.00
	1	17/26	65.38	9/26	34.62	4/26	15.38
	2	11/26	42.31	8/26	30.77	15/26	57.69
	3	10/26	38.46	10/26	38.46	17/26	65.38
	TOTAL	44/87	50.57	30/87	34.48	36/87	41.38
FEED	1	22/26	84.62	17/26	65.38	26/26	100.00
	2	20/25	80.00	18/25	72.00	25/25	100.00
	3	20/25	80.00	19/25	76.00	25/25	100.00
	TOTAL	62/76	81.58	54/76	71.05	76/76	100.00
BED	0	9/10	90.00	8/10	80.00	3/10	30.00
	1	18/26	69.23	22/26	84.62	15/26	57.69
	2	18/26	69.23	24/26	92.31	14/26	53.85
	3	18/25	72.00	23/25	92.00	13/25	52.00
	TOTAL	63/87	72.41	77/87	88.51	45/87	51.72
**TOTAL**		169/250	67.60	161	64.40	157	62.80

References:

EUCAST, 2025. European Committee on Antimicrobial Susceptibility Testing. Breakpoint tables for interpretation of MICs for antifungal agents (No. Version 12).

Gaspar, D.P., Faria, V., Gonçalves, L.M.D., Taboada, P., Remuñán-López, C., Almeida, A.J., 2016. Rifabutin-loaded solid lipid nanoparticles for inhaled antitubercular therapy: Physicochemical and in vitro studies. Int. J. Pharm. 497, 199–209. https://doi.org/10.1016/j.ijpharm.2015.11.050

Guinea, J., Meletiadis, J., Arikan S, Muehlethaler K, Kahlmeter G, Arendrup MC, 2022. Method for the determination of broth dilution minimum inhibitory concentrations of antifungal agents for conidia forming moulds.

Guinea, J., Verweij, P.E., Meletiadis, J., Mouton, J.W., Barchiesi, F., Arendrup, M.C., Arendrup, M.C., Arikan-Akdagli, S., Barchiesi, F., Castanheira, M., et al., 2019. How to: EUCAST recommendations on the screening procedure E.Def 10.1 for the detection of azole resistance in Aspergillus fumigatus isolates using four-well azole-containing agar plates. Clin. Microbiol. Infect. 25, 681–687. https://doi.org/10.1016/j.cmi.2018.09.008

Serrano-Lobo, J., Reigadas, E., Muñoz, P., Escribano, P., Guinea, Jesús, Romera, W.S.-Y., Sánchez-Gómez, J.A., Lozano, I.G., Tejero-García, R., et al.,, 2024. Azole resistance screening in *Aspergillus fumigatus sensu stricto* using the azole-containing agar method (EUCAST E.Def 10.2): conidial suspension filtration and inoculum adjustment before inoculum preparation may not be needed. J. Clin. Microbiol. 62, e00369-24. https://doi.org/10.1128/jcm.00369-24
